# Fibrotic liver microenvironment promotes Dll4 and SDF-1-dependent T-cell lineage development

**DOI:** 10.1038/s41419-019-1630-1

**Published:** 2019-06-05

**Authors:** Zheng Gong, Bingxue Shang, Yunpeng Chu, Xiaodong Chen, Qing Li, Keli Liu, Yongjing Chen, Yin Huang, Yanyan Han, Qianwen Shang, Zhiyuan Zheng, Lin Song, Yanan Li, Rui Liu, Chenchang Xu, Xiaoren Zhang, Baochi Liu, Luowei Wang, Changshun Shao, Ying Wang, Yufang Shi

**Affiliations:** 1 The First Affiliated Hospital of Soochow University, Institutes for Translational Medicine, State Key Laboratory of Radiation Medicine and Protection, Key Laboratory of Stem Cells and Medical Biomaterials of Jiangsu Province, Soochow University Medical College, Suzhou, China; 2 Key Laboratory of Tissue Microenvironment and Tumor, Institute of Health Sciences, Shanghai Institutes for Biological Sciences, Chinese Academy of Sciences, University of Chinese Academy of Sciences, Shanghai, China; 3 Department of Surgery, Shanghai Public Health Clinical Center Fudan University, Shanghai, China; 4 Department of Gastroenterology, Changhai Hospital, Second Military Medical University, Shanghai, China

**Keywords:** Bone marrow transplantation, T cells, Stem-cell differentiation

## Abstract

The reconstitution of the T-cell repertoire and quantity is a major challenge in the clinical management of HIV infection/AIDS, cancer, and aging-associated diseases. We previously showed that autologous bone marrow transfusion (BMT) via the hepatic portal vein could effectively restore CD4^+^ T-cell count in AIDS patients also suffering from decompensated liver cirrhosis. In the current study, we characterized T-cell reconstitution in a mouse model of liver fibrosis induced by CCl_4_ and found that T-cell reconstitution after BMT via hepatic portal vein was also greatly enhanced. The expression of *Dll4* (Delta-like 4), which plays an important role in T-cell progenitor expansion, was elevated in hepatocytes of fibrotic livers when compared to normal livers. This upregulation of *Dll4* expression was found to be induced by TNFα in an NFκB-dependent manner. Liver fibroblasts transfected with Dll4 (LF-Dll4) also gained the capacity to promote T-cell lineage development from hematopoietic stem cells (HSCs), resulting in the generation of DN2 (CD4 and CD8 DN 2) and DN3 T-cell progenitors in vitro, which underwent a normal maturation program when adoptively transferred into *Rag-2* deficient hosts. We also demonstrated a pivotal role of SDF-1 produced by primary liver fibroblasts (primary LF) in T-lineage differentiation from HSCs. These results suggest that Dll4 and SDF-1 in fibrotic liver microenvironment could promote extrathymic T-cell lineage development. These results expand our knowledge of T-cell development and reconstitution under pathological conditions.

## Introduction

Reconstitution of T cells is a major clinical challenge in patients suffering from HIV/AIDS, cancer, and aging-associated diseases. T-cell deficiency is a cardinal feature of aging or HIV infection and underlies susceptibility to infectious microorganisms and malignancies^1,2^. In cancer patients, chemotherapy or preparative regimens for bone marrow transplantation result in severe and protracted lymphopenia, and the recovery of T-cell populations is often delayed compared to that of myeloid, NK, or B cells^3^. Moreover, T-cell function often remains compromised even after lymphocyte numbers are restored to normal level, likely due to a reduced T-cell clonal diversity^4–6^. In AIDS patients, although the viral loads in peripheral blood can be kept under control after antiretroviral therapy, their health conditions can still deteriorate with dramatically decreased CD4^+^ T-cell count. Stable reconstitution of CD4^+^ T-cell number and functional clonality, with complete suppression of viral replication and elimination of viral reservoirs, has been the ultimate goal of AIDS therapy^7^. We previously showed that administration of autologous bone marrow transfusion (BMT) via the hepatic portal vein could effectively restore CD4^+^ T-cell count in AIDS patients who were also suffering from decompensated liver cirrhosis^8,9^. This observation indicates a possibility that the cirrhotic liver microenvironment may possess lymphopoiesis supportive activity that can direct autologous hematopoietic stem/progenitor cells to undergo differentiation toward early T lineage.

T-cell development occurs in multiple discrete steps and is controlled by complex regulatory mechanisms^10^. Thymocytes can be divided into the categories of double-negative (DN), immature double-positive (DP), and mature single-positive (SP) cells based on their expression of CD4 and/or CD8^11^. In the mouse, DN thymocytes are typically classified, according to the surface expression of CD25 and CD44 or CD117 (c-kit), into DN1–DN4 subsets. CD4^+^CD8^+^ DP thymocytes are subjected to positive and negative selection in the thymus^12^. Following these selections, mature, CD4 or CD8 SP thymocytes are exported from the thymus to establish the pool of self-restricted and functional T cells in the periphery^13^.

In the current study, we showed that liver fibrosis induced by CCl_4_ in a murine model could promote T-cell reconstitution after BMT via hepatic portal vein. Surprisingly, Dll4, an important factor in T-cell progenitor development, was upregulated in hepatocytes of the fibrotic tissues. We also showed that liver fibroblasts expressing Delta-like 4 (LF-Dll4) gained the capacity to induce a normal program of early T-cell development from hematopoietic stem cells (HSCs). These DN2 and DN3 T-cell progenitors generated in vitro were found to follow a normal maturation program in the thymus of *Rag-2* deficient hosts when adoptively transferred. We also demonstrated a pivotal role of stromal cell-derived factor-1 (SDF-1)/chemokine CXC chemokine ligand 12 (CXCL12)/pre-B-cell growth stimulating factor in primary LF-Dll4 in directing HSC differentiation into T lineage. These results suggested that Dll4 and SDF-1 in the fibrotic liver microenvironment promote early T-cell development and maturation.

## Results

### Enhanced T-cell reconstitution by BMT in mice suffering from CCl_4_-induced liver fibrosis

**Fig. 1:**
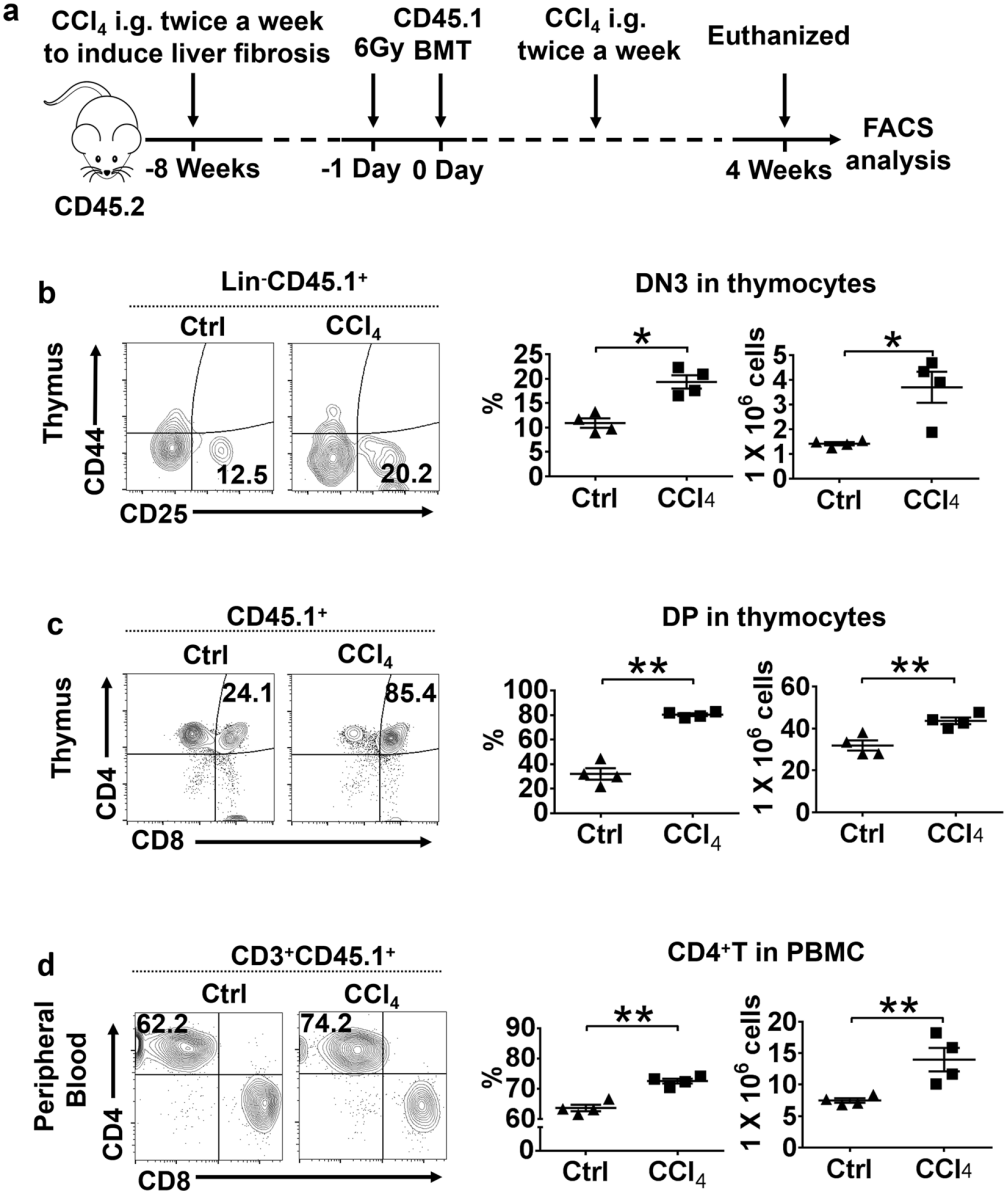
Liver fibrosis induced by CCl_4_ promotes T-cell reconstitution. **a** Schematic representation of the experimental procedures using CCl_4_-induced liver fibrosis followed by BMT in a mouse model. **b** Flow cytometric analysis for the expression of CD25 and CD44 on thymocyte for DN1–DN4 stages of the T-cell development in the thymus on day 28 after CD45.1 BMT through the hepatic portal vein. **c** Flow cytometric analysis for the expression of CD4 and CD8 in thymocytes for the DP and SP stages of T-cell development, on day 28 after CD45.1 BMT. **d** Flow cytometric analysis for the expression of CD4 and CD8 on PBMCs for CD4^+^ and CD8^+^ T cells in peripheral blood on day 28 after CD45.1 BMT. The results are presented as mean ± S.E.M. Statistical significance was determined by Student’s *t* test. Significance between samples is indicated in the figures as follows: *P < 0.05; **P < 0.01

We previously reported that autologous BMT via the hepatic portal vein could effectively reconstitute peripheral CD4^+^ T-cell counts and hepatic function in splenectomized AIDS patients with decompensated liver cirrhosis^8,9^. To recapitulate this observation in an experimental setting, we induced liver fibrosis with CCl_4_ in combination with splenectomy in CD45.2/C57BL/6J mice and examined the subsequent T-cell reconstitution. Splenectomy, by spleen ligation and removal, was carried out immediately after CD45.1/C57BL/6J bone marrow cells (BMCs) were transplanted in control and CCl_4_ treated mice (Fig. 1a). Flow cytometry showed significant differences of T-lineage populations between the CCl_4_-treated and the control groups in the thymus and peripheral blood 28 days after BMT. Donor cells were identified by CD45.1. CD44^+^CD25^–^, CD44^+^CD25^+^, CD44^−^CD25^+^, and CD44^−^CD25^−^ marked DN1–DN4 T-lineage cell populations, respectively. CD4^+^CD8^−^, CD4^−^CD8^+^, CD4^+^CD8^+^, and CD4^−^CD8^−^ in the thymus indicated CD4SP, CD8SP, DP, and DN T-lineage populations, respectively. The percentages and absolute numbers of both DN3 and DP cells were greater in the CCl_4_-treated group than in the control group (Fig. 1b, c). In peripheral blood, a noticeable increase in the percentage and absolute numbers of CD4^+^ T-cell population was also observed in the CCl_4_-treated group over the control group (Fig. 1d). In contrast, such increase was not observed in the liver (Supplementary Fig. 1). The endogenous cells in the recipient mice (CD45.2^+^) appeared to be unaffected by fibrosis after irradiation and BMT (Supplementary Fig. 2a–c). CCl_4_ treatment alone also had no effect on the total number of thymocytes (Supplementary Fig. 3a, b).

**Fig. 2:**
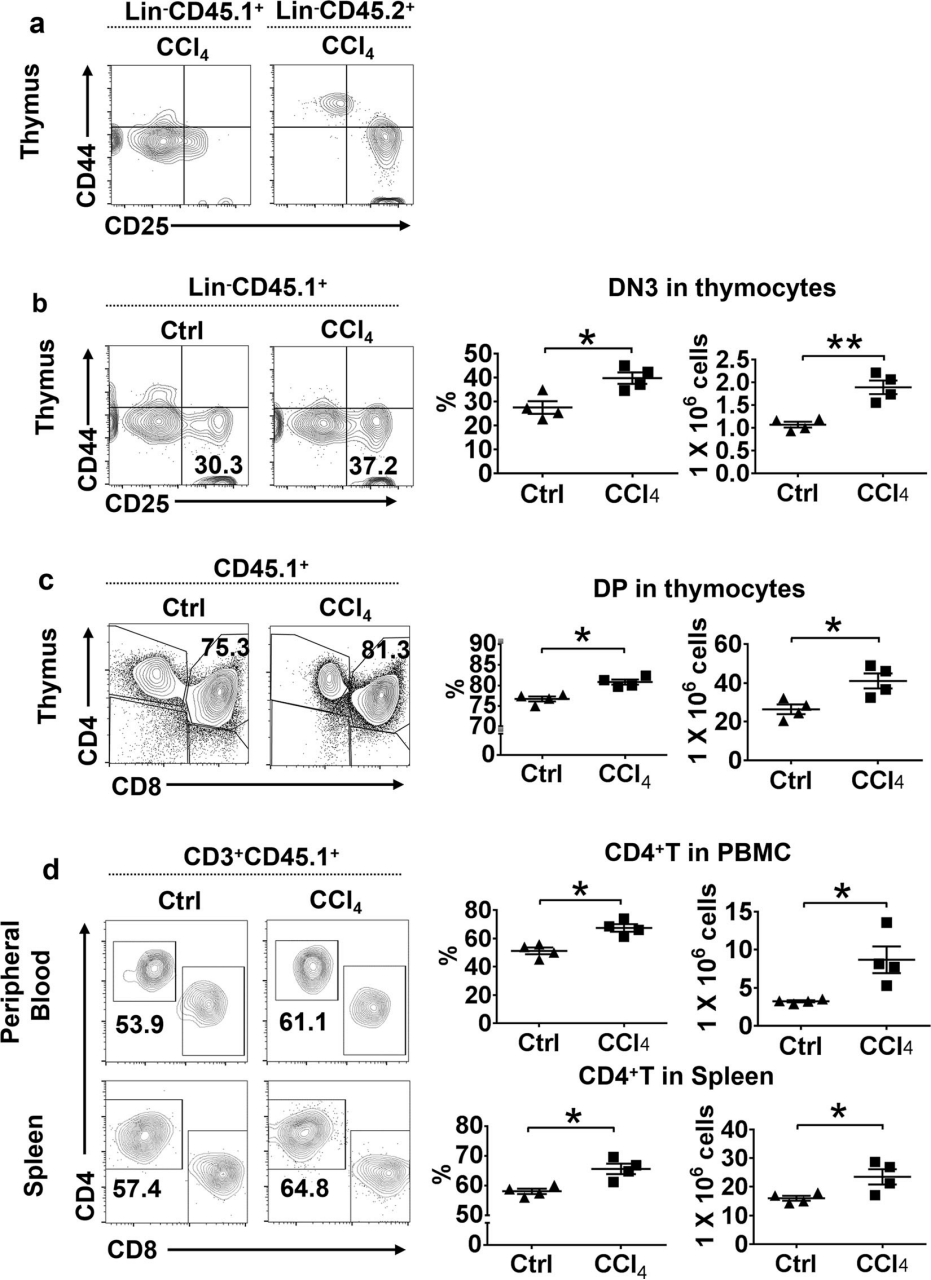
Analysis of engraftment and differentiation of BMCs in *Rag2*^−/−^ mice. **a** Flow cytometric analysis for the expression of CD25 and CD44 in thymocytes on day 28 after CD45.1 BMT through the hepatic portal vein. Differentiation of CD45.1 donor cells can be detected (left). **b** Flow cytometric analysis for the expression of CD25 and CD44 on thymocytes for DN1–DN4 stages of T-cell development in thymus of *Rag2*^–/–^ mice with and without CCl_4_ treatment on day 28 after CD45.1 BMT. **c** Flow cytometric analysis for the expression of CD4 and CD8 in thymocytes for DP and SP stages of T-cell development in the thymus of *Rag2*^−/−^ mice with or without CCl_4_ treatment on day 28 after CD45.1 BMT. **d** Flow cytometric analysis for the expression of CD4 and CD8 in lymphocytes for CD4^+^ and CD8^+^T cells in peripheral blood and spleen of *Rag2*^−/−^ mice with or without CCl_4_ treatment on day 28 after CD45.1 BMT. The results are presented as mean ± S.E.M. Statistical significance was determined by Student’s *t* test. Significance between samples is indicated in the figures as follows: *P < 0.05; **P < 0.01

To eliminate possible influences by host T cells and splenectomy, which may affect donor T-cell reconstitution in recipient mice after BMT, on T-lymphopoiesis under the fibrotic microenvironment, we also transplanted CD45.1 congenic BMCs into *Rag2*^–/–^ CD45.2/C57BL/6J hosts without splenectomy and obtained similar results (Fig. 2a–d). Flow cytometric analysis revealed significant differences of T-lineage-cell populations between the CCl_4_-treated and control groups in the thymus, peripheral blood, and spleen. The DN3 and DP T-lineage populations were greater in the CCl_4_-treated group than in the control group (Fig. 2b, c). In the CCl_4_-treated group, CD4^+^ T-cell percentage and absolute numbers were also higher in both peripheral blood and splenic CD3^+^ T-cell subsets than in the control group (Fig. 2d).

These data suggest that the mouse model based on CCl_4_ treatment and splenectomy could recapitulate some of the clinical features manifested in autologous bone marrow-treated AIDS patients with fibrotic livers, and the fibrotic liver microenvironment may play an important role in T-cell reconstitution after BMT via the hepatic portal vein.

### Dll4 expressed in hepatocytes of fibrotic liver promotes T-cell development

**Fig. 3:**
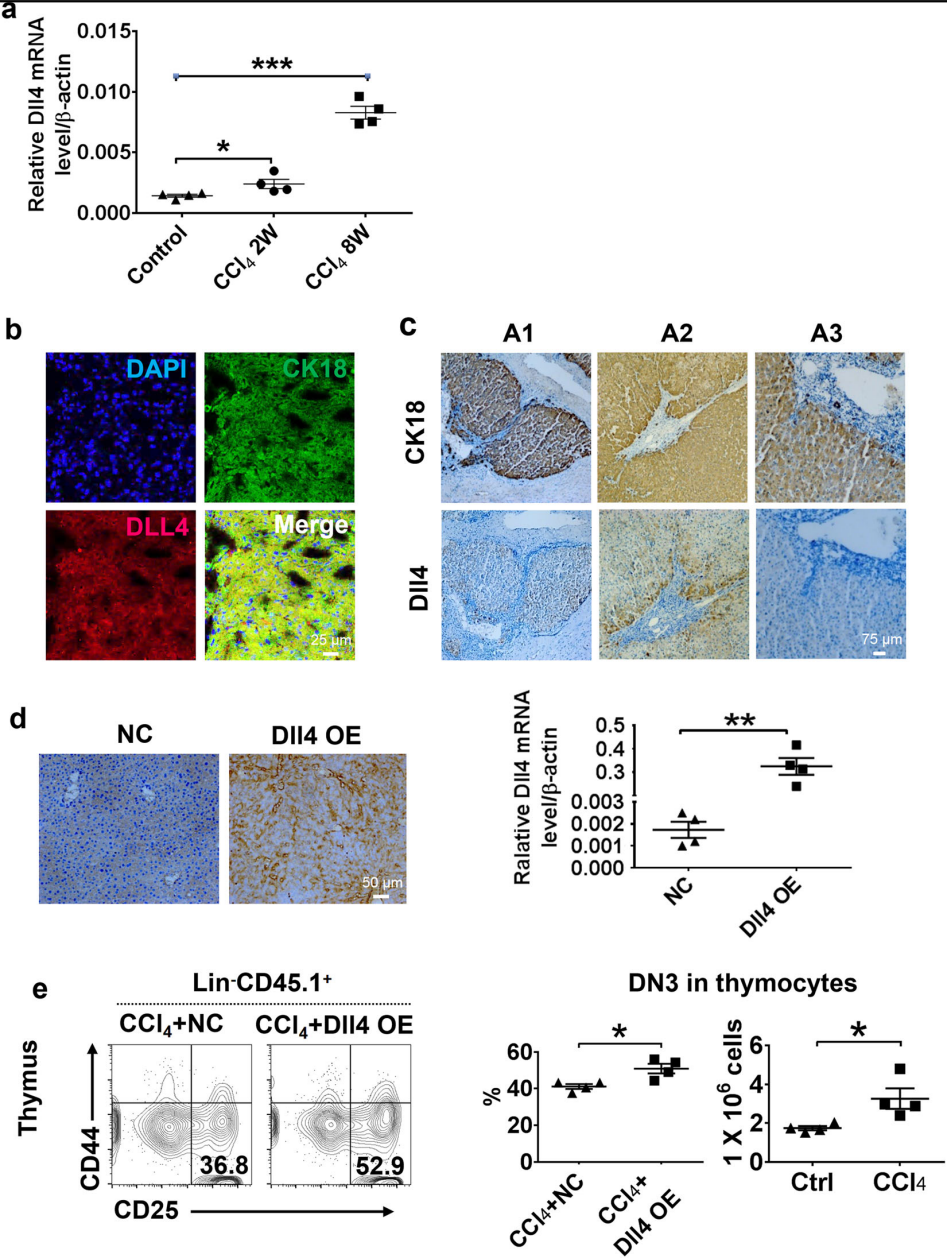
Elevated Dll4 expression in hepatocytes of fibrotic liver promotes T-cell lineage development. **a** Liver tissues from mice suffering from liver cirrhosis induced by CCl_4_ were collected and qRT-PCR was performed on total RNA to determine the level of *Dll4* mRNA. *P < 0.05; ***P *<* 0.001 compared to the control. **b** The cell types and the levels of Dll4 expression in fibrotic mouse liver tissue were examined by immunofluorescence, using Dll4 and CK18-specifc antibodies. **c** Serial sections of liver tissue from different patients (A1–A3) suffering from AIDS and different degrees of liver cirrhosis, and immunohistochemistry analysis were employed to examine the cell types with Dll4 expression and the level of Dll4 expression change in different patients, using Dll4 and CK18-specific antibodies. **d** Generation of a mouse model with ectopic Dll4 expression in liver tissue by adenoviral transfection; immunohistochemistry analysis was used to examine the level of Dll4 expression between the empty vector control group and the Dll4 overexpression group. Total RNA was harvested and the *Dll4* mRNA level was analyzed by qRT-PCR. **P < 0.01 compared to the empty vector control. **e** The expression levels of CD25 and CD44 on thymocytes were analyzed by flow cytometry in mouse liver tissues from mice with and without Dll4-overexpressing vector treatment in mice suffering from CCl_4_-induced liver fibrosis on day 28 after CD45.1 BMT through the hepatic portal vein. The results are presented as mean ± S.E.M. Statistical significance was determined by Student’s *t* test. Significance between samples is indicated in the figures as follows: *P < 0.05; **P < 0.01; ***P < 0.001

Since Notch signaling is essential for T-lymphoid commitment and is required for the progression through the DN stage^14–18^, we hypothesized that Notch signaling may contribute to the enhanced CD4^+^ T-cell development in CCl_4_-induced liver fibrosis. Indeed, we found that the mammalian Notch ligand Dll4 expression was gradually increased in the liver with increased duration of CCl_4_ treatment (Fig. 3a). To determine the cellular source of Dll4 in the fibrotic liver tissue, we stained liver sections of CCl_4_-treated mice by immunofluorescence (Fig. 3b and Supplementary Fig. 4a), and found that Dll4 was expressed by hepatocytes (CK18^+^), but not by endothelial (CD31^+^) or myofibroblast cells (αSMA^+^) in fibrotic liver tissue. Interestingly, in human fibrotic liver tissues, immunohistochemistry staining showed that Dll4 colocalized with hepatocytes marked by CK18, but not with endothelial cells and myofibroblasts (Fig. 3c and Supplementary Fig. 4b). We also isolated mouse primary LF and primary hepatocytes from CCl_4_-treated mice, and found that *Dll4* mRNA was highly expressed in primary hepatocytes, but was barely detectable in primary LF cells (Supplementary Fig. 4c). The expression of *Dll4* was low in hepatocytes of control mice. The data indicate that Dll4 was selectively upregulated in hepatocytes of fibrotic liver.

To further determine the role of Dll4 in T-cell reconstitution in vivo, we used adenoviral expression vectors to generate mice with ectopic Dll4 expression in the liver tissue (Fig. 3d). We performed flow cytometry to quantify T lineage in CCl_4_-treated Dll4 overexpression (OE) and normal control (NC) mice after BMT via the hepatic portal vein. Dll4 ectopic expression in the liver resulted in a marked increase in DN3 percentage and absolute numbers in the thymus (Fig. 3e). However, Dll4 OE alone had no effect on the percentage and total number of thymocytes in the thymus, and lymphocytes in peripheral blood and spleen compared with control (NC) (Supplementary Fig. 5a–c). It should be noted that no increased Dll4 expression was detected in thymus (Supplementary Fig. 6a), refuting the possibility that the increase in the DN3 population was due to an alteration in thymus function.

Together, these results demonstrated that liver fibrosis could upregulate Dll4 expression in hepatocytes, and this expression of Dll4 contributes to the early stage T-cell development by promoting T-cell progenitor development.

### Induction of Dll4 in hepatocytes is partially NFκB-dependent

**Fig. 4:**
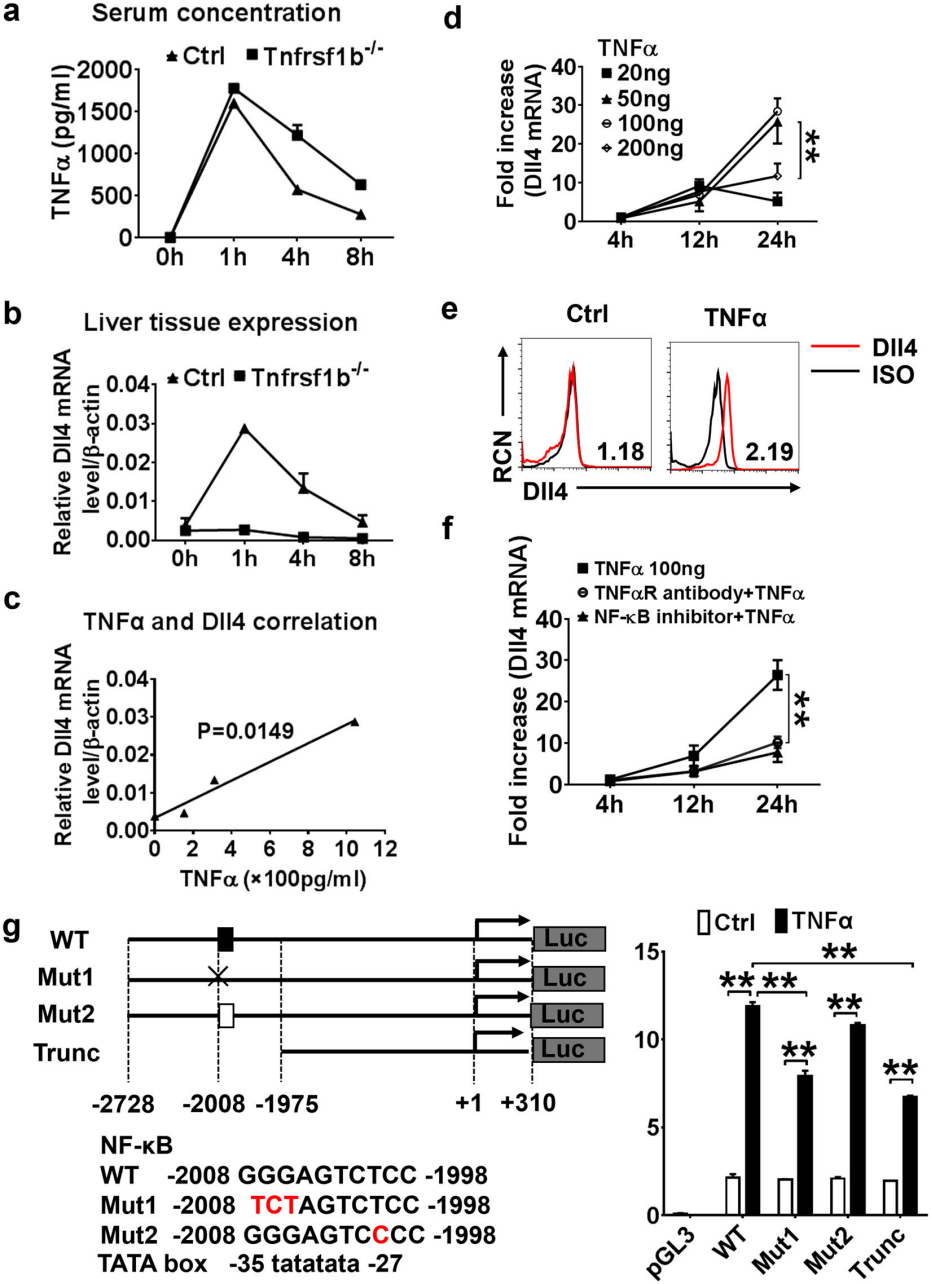
Induction of Dll4 in fibrotic hepatocytes through an NFκB-dependent mechanism. **a** Serum TNFα concentrations in normal C57BL/6J mice and *Tnfrsf1b*^−/−^ mice were detected by ELISA, after LPS were injected by IP. **b** Liver tissues were collected and mRNA was harvested for analysis of Dll4 expression by qRT-PCR. **c** Pearson correlation analysis of the serum TNFα concentrations and Dll4 mRNA levels in the liver. **d** Primary hepatocytes were treated with TNFα at different concentrations for the indicated times and mRNA was harvested for qRT-PCR analysis. **P < 0.01. **e** Primary hepatocytes were rested or treated with TNFα (50 ng/ml) for 24 h and harvested for flow cytometry analysis using either an isotype control or a Dll4-APC-specific antibody. **f** Primary hepatocytes were treated with TNFα (100 ng/ml) for the indicated times in the presence of control antibody, a blocking antibody to TNFαR, or NF-κB inhibitor JSH-23, and mRNA was harvested for qRT-PCR analysis of Dll4 expression. **P < 0.01. **g** The Dll4 promoter containing an NF-κB binding element responds to TNFα. The mouse Dll4 promoter was cloned upstream of the luciferase gene in pGL3-basic vector. The WT and Mut with different mutant NF-κB sites are indicated. A truncated version of the promoter that lacks the NFκB binding site was also generated. Primary hepatocytes were transfected with the control (pGL3-basic) vector, the reporter vectors containing the WT Dll4 promoter, or the reporter vectors containing the Dll4 promoter with mutant NFκB binding site. Cells were transfected and rested 48 h and then treated for 8 h with TNFα (100 ng/ml) before lysis and luciferase assay. The results are presented as mean ± S.E.M. Statistical significance was determined by Student’s *t* test. Significance between samples is indicated in the figures as follows: **P < 0.01

We next determined whether the increased expression of Dll4 in fibrotic liver is associated with increased inflammation. We employed *Tnfrsf1b*^−/−^ mice, which is defective in response to bacterial endotoxin, LPS. Thus, wild-type (WT) and *Tnfrsf1b*^−/−^ C57BL/6J mice were injected with lethal doses of LPS intraperitoneally, euthanized at different time points, and their liver tissues were then assayed for *Dll4* mRNA expression. We found that the induction of *Dll4* mRNA expression in WT mice was rapid and reached peak expression at 1 h post LPS administration, and it was strongly correlated with serum TNFα concentration (Fig. 4a–c). In sharp contrast, no Dll4 induction was detected in *Tnfrsf1b*^−/−^ mice after LPS treatment, though there was a remarkable induction of the TNFα (Fig. 4a, b). This result indicates that TNFα signaling pathway is critical for the induction of Dll4 in hepatocytes.

The levels of TNFα concentration in CCl_4_-treated mice were significantly and persistently higher than those in NC mice (Supplementary Fig. 7a). To further determine the role of TNFα and its signaling pathway in inducing the expression of Dll4 in hepatocytes, we isolated primary hepatocytes by two-step collagenase perfusion of normal C57BL/6J liver tissues (Supplementary Fig. 8a). Primary hepatocytes treated with LPS and different cytokines: IFNα, IL-3, IL-4, IL-12, IL-17, M-CSF, IFNγ, TNFα, IL-1β or IL-15, and Dll4 mRNA expression was assessed by qRT-PCR. Among all treatments, TNFα treatment strongly induced Dll4 expression at mRNA level (Supplementary Fig. 8b). TNFα at 50 and 100 ng/ml showed the highest induction of *Dll4* expression (Fig. 4d). Assay of cell surface protein expression of Dll4 by flow cytometry also revealed a 2.19-fold increase by TNFα (Fig. 4e). We also used TNFαR-specific blocking antibodies at 5 μg/ml or NFκB inhibitor JSH-23 to determine whether TNFαR and NFκB are involved in the induction of *Dll4* mRNA. We found that blockade of TNFαR or inhibition of the NFκB pathway could significantly attenuate Dll4 induction by TNFα (Fig. 4f), *p*-value < 0.01. These data suggested that TNFα signals act through TNFαR and the NFκB pathway to induce *Dll4* expression in hepatocytes.

We then analyzed the mouse *Dll4* promoter using the TRANSFAC database and identified potential binding sites for known TNFα inducible transcription factors. Of particular interest was a perfect consensus NFκB site at −2008 to −1998 bp upstream of the transcription start site (TSS) (Fig. 4g). We PCR-amplified a 3.04-kb fragment of the mouse promoter region and cloned it into the pGL3-basic reporter vector. This construct, referred to as WT, occurs 2728 bp upstream and 325 bp downstream of the TSS. We also constructed Mut1 version possessing a 3-bp mutation in the putative NFκB site of the WT promoter designed to block binding of NFκB proteins (GGGAGTCTCC to ***TCT***AGTCTCC), and Mut2 version with 1-bp mutation in the putative NFκB site of the WT promoter change to another kind of NFκB binding site sequence (GGGAGTCTCC to TCTAGTC***C***CC), and then generated a truncated version of the promoter (−1975 to +58 bp) lacking the NFκB site. When transfected into primary hepatocytes and assayed for luciferase activity in control and TNFα-treated cells, the Mut2 and WT promoter was strongly responsive to TNFα with 5.19- and 5.53-fold increase over the control, whereas the truncated promoter had only a 3.43-fold increase over the control. Mut1 promoter responded to TNFα with a 3.92-fold increase over the control. These results indicate that induction of Dll4 in hepatocytes is partially NFκB-dependent.

### HSCs cocultured with Dll4-expressing hepatocytes and liver fibroblasts undergo T lymphopoiesis

**Fig. 5:**
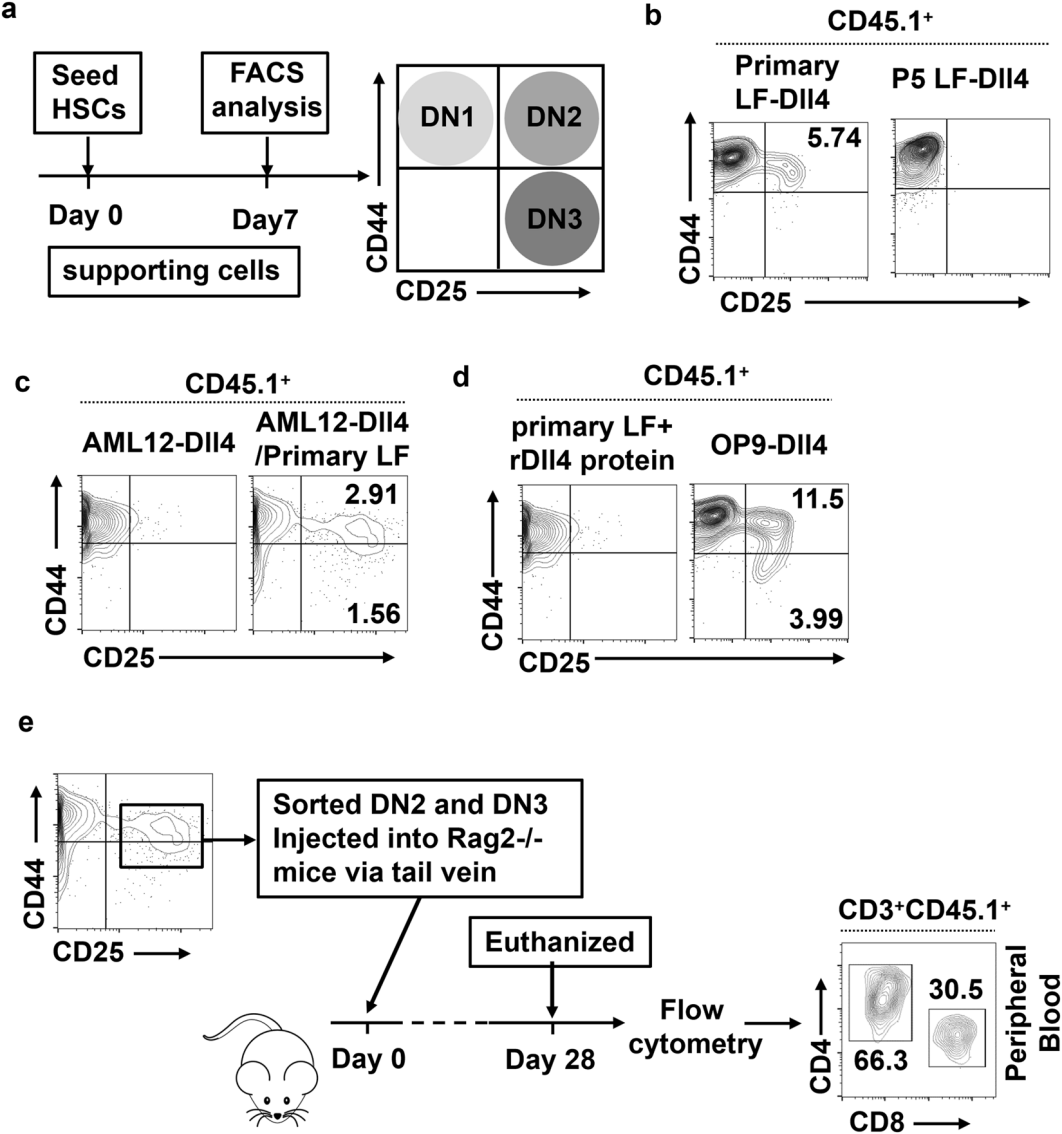
HSCs cocultured with Dll4-expressing hepatocytes and liver fibroblasts undergo T lymphopoiesis. **a** Schematic representation of the experimental procedures for assessing the development of T-cell lineage in the coculture of HSCs and cells overexpressing Dll4. **b** Sorted HSCs (Lin^−^Sca1^+^CD117^+^) derived from the bone marrow of CD45.1/C57BL/6J mice were cocultured with primary LF-Dll4 or P5 LF-Dll4 cells. HSCs were harvested after 7 days and analyzed by flow cytometry for the surface expression of CD25 and CD44 as indicated. Cells were gated as live (DAPI^−^) and CD45.1^+^. Data are representative of at least three independent experiments. **c** The expression of CD25 and CD44 was analyzed by FACS on day 7 after HSCs cocultured with AML12-Dll4 or AML12-Dll4 (1:1 ratio) mixed with primary LF. **d** The expression of CD25 and CD44 was analyzed by flow cytometry on day 7 after HSCs cocultured with the indicated cell lines. **e** Analysis of engraftment and differentiation of in vitro-derived progenitor T-cell subsets in *Rag2*^−/−^ mice. HSCs were differentiated for 7 days on primary LF-Dll4 cells, and the DN2 (CD25^+^CD44^+^) and DN3 (CD25^+^CD44^−^) subsets were sorted. Cells (3–5 × 10^5^) were subsequently injected into the tail vein of *Rag2*^−/−^ mice and the peripheral blood of injected mice was harvested and analyzed for cell surface expression of CD4 and CD8 (*n* = 5) on day 28

The OP9 cell line was established from newborn calvaria of the (C57BL/6J × C3H) F2-op/op mouse deficient in M-CSF^19^. OP9-DL1 and OP9-DL4 cells have been frequently used to study the differentiation of mouse and human T lymphocytes from different sources of stem cells in vitro^20–22^. Since OP9 cells or thymic stromal cells (TSCs)^23^ ectopically expressing Dll4 were sufficient for the induction and sustenance of T-cell development in vitro (Supplementary Fig. 9a, b), whereas ectopic expression of Dll4 in NIH/3T3 and mesenchymal stem cells (MSCs) failed to drive T-lineage development (Supplementary Fig. 9c), we hypothesized that fibrotic liver-associated hepatocytes might have only produced Dll4 to activate the Notch signaling pathway in HSCs, and liver fibroblast cells may have provided the other factors required for HSCs to further differentiate into T lineage. To determine whether the liver fibroblast cells expressing Dll4 ligands can support the generation of T cells, we isolated primary LF from fibrotic liver and also obtained pure liver fibroblast with αSMA expression by passaging for five generations (P5 LF) (Supplementary Fig. 10a). We transduced primary and P5 liver fibroblasts with lentiviral puro-Dll4 (LF-Dll4) (Supplementary Fig. 11a), expanded the cells in medium containing puromycin, and examined their ability to initiate and support the differentiation of early T lineage in vitro. To achieve this, we cocultured HSCs with primary LF-Dll4, P5 LF-Dll4, or OP9-Dll4 and analyzed the differentiation of CD45.1^+^ cells on day 7 (Fig. 5a). The primary LF-Dll4 cells, like OP9, could readily support the generation of early T-lineage progenitors. In contrast, P5 LF-Dll4 cells did not possess the ability to promote T-cell development. On day 7, primary LF-Dll4 cocultures contained 5.74% CD25^+^ early T-lineage cells (Fig. 5b). However, this effect of primary LF-Dll4 was inhibited by Notch signaling pathway inhibitor DAPT (*N*-[*N*-(3,5-difluorophenacetyl-L-alanyl)]-S-phenylglycine t-butyl ester) (Supplementary Fig. 12a). The primary LF cells derived from fibrotic livers were not capable of promoting T-cell development (Supplementary Fig. 12b). AML12 (alpha mouse liver 12) was a hepatocyte cell line established from CD1 strain. To mimic hepatic fibrotic microenvironment, we established AML12 cells ectopically expressing Dll4 (AML12-Dll4). HSCs cocultured with AML12-Dll4 failed to produce T lineage. However, when HSCs were seeded over AML12-Dll4/primary LF cells mixed in 1:1 ratio, they were found to give rise to early T-cell progenitors (Fig. 5c). This result indicates that Dll4 expressed by hepatocytes alone is not sufficient to drive T-cell development. We then investigated whether soluble Dll4 could also promote T-cell development by adding rDll4 protein into the culture medium of HSCs/primary LF cells coculture system. As shown in Fig. 5d, soluble rDll4 failed to promote T-lineage development.

We next determined whether T progenitors obtained from these cultures could also give rise to SPs in vivo. To this end, CD45.1^+^ DN2 (CD44^+^CD25^+^) and CD45.1^+^ DN3 (CD44^−^CD25^+^) cells were sorted by CD25 microbeads from CD45.1^+^ HSCs/AML12-Dll4/primary LF cocultured for 7 days (Fig. 5e), and injected via tail vein into 8-week-old CD45.2^+^ *Rag2*^−/−^ mice. Flow cytometric analysis showed that, 4 weeks after transfusion, CD45.1^+^ CD8 SPs and CD45.1^+^ CD4 SPs were readily detected in the peripheral blood of the CD45.2^+^ *Rag2*^−/−^ recipient mice (Fig. 5e). These results indicate that progenitors generated from HSCs/ AML12-Dll4/primary LF cocultures could be used for in vivo applications.

These data suggested that Dll4 produced by hepatocytes was important for T-cell development, but liver fibroblasts were also needed for the provision of the other factors required for inducing HSCs to differentiate into T lineage.

### SDF-1 plays an essential role in T-lineage development from HSCs

**Fig. 6:**
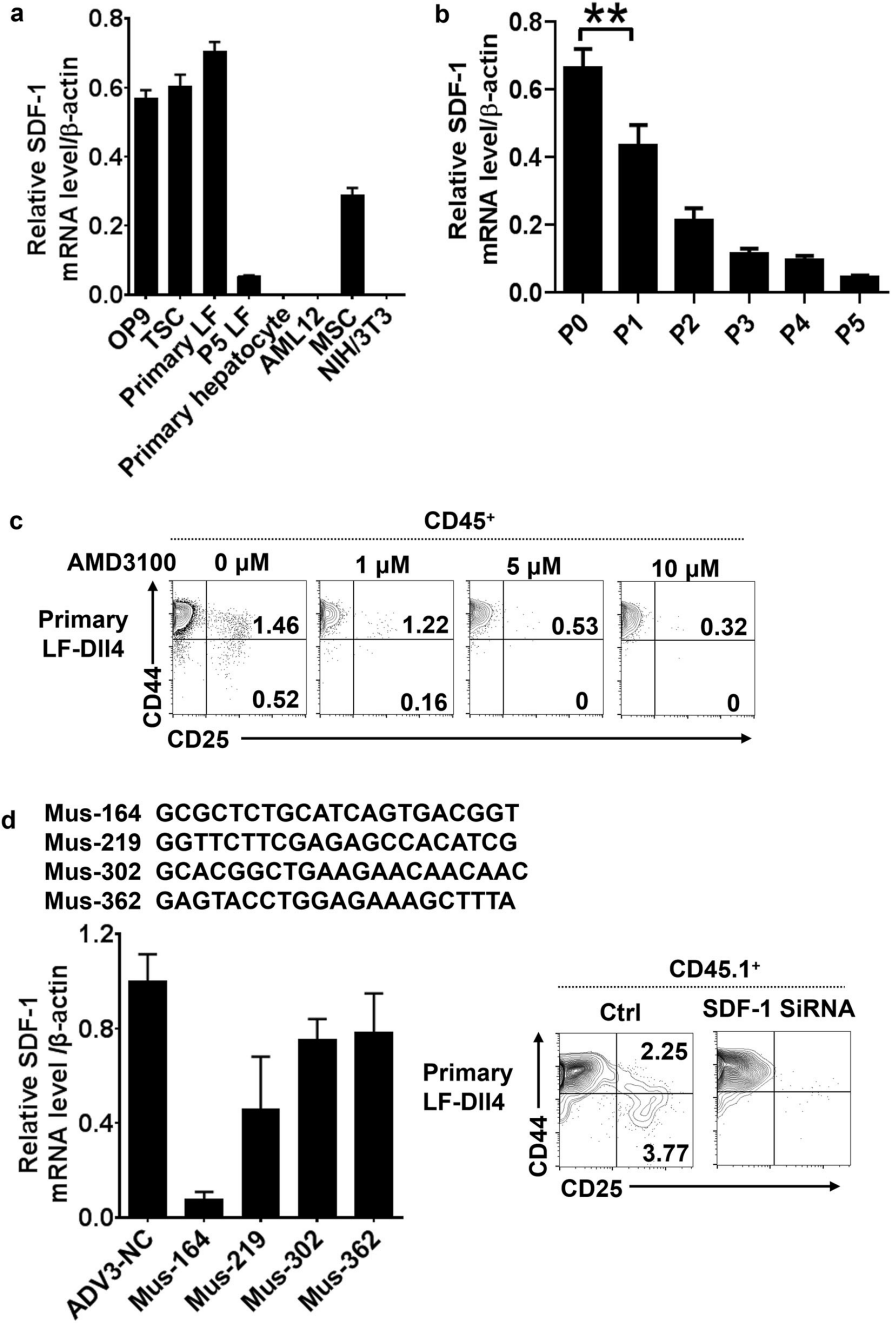
SDF-1 expression in primary LF cells plays an essential role in lymphopoiesis in vitro. **a** Different cultured cells were collected and mRNA was analyzed for the level of SDF-1 expression by qRT-PCR. **b** qRT-PCR detected SDF-1 expression in 0–5 successive generations of primary liver fibroblast cells. **P < 0.01. **c** The expression levels of CD25 and CD44 were analyzed by flow cytometry on day 7 after coculture of HSCs with primary LF-Dll4 with or without different concentration of AMD3100. **d** Generation of adenovirus carrying SDF-1 siRNA and transfection of primary liver fibroblast cells. Transfected cells were rested for 48 h and the level of SDF-1 expression after adenovirus transfection was detected by qRT-PCR, and flow cytometric analysis of the expression of CD25 and CD44 on day 7 after HSCs co-cultured with SDF-1-depleted primary LF-Dll4 or control LF-Dll4

The ability of primary LF-Dll4 to promote T lymphopoiesis was lost after passaging for five generations. We wondered if some factors differently expressed between primary LF and P5 LF were responsible for the differential effect. Genes encoding various inflammatory factors and chemotactic/growth factors were analyzed by qPCR. SDF-1 was found to be expressed at a much higher level in the cells that are able to support T-lineage development, OP9, TSC, and primary LF, than in nonfunctional cells, including P5 LF, NIH/3T3, AML12, and primary hepatocytes (Fig. 6a). Consistently, we observed that the level of SDF-1 in primary LF cells was gradually downregulated with increasing passages (Fig. 6b). Taken together, these data suggest that SDF-1 derived from primary LF could positively regulate the differentiation of HSCs to the T lineage.

To determine that the promotion of T lymphopoiesis by the primary LF-Dll4 was dependent on SDF-1 signaling in our coculture system, we used AMD3100 (Sigma), a specific CXCR4 antagonist that inhibits the binding and function of SDF-1 with high affinity and potency^24^, in the coculture system. As shown in Fig. 6c, the percentages of DN2 and DN3 were gradually decreased with increasing AMD3100 concentrations (Fig. 6c).

We further determined the role of SDF-1 in promoting HSC differentiation to T lineage by depleting SDF-1 expression in primary LF-Dll4 using adenoviral transfection of SDF-1 small interfering RNA (siRNA). We successfully silenced SDF-1 expression in primary LF cells (Fig. 6d). These SDF-1-deficient primary LF cells were unable to support T lymphopoiesis.

## Discussion

Liver has been shown to be a major site for extrathymic T-cell development. Previous studies have revealed that bone marrow derived CD117^+^ precursors are able to migrate to and lodge in the liver during fetal development, as well as in adulthood^25^. CD117^+^ progenitor cells have also been shown to be capable of giving rise to extrathymic T lymphocytes under certain pathological conditions, including infection, autoimmune disease, and malignancy^26–28^. We here showed that mice with liver fibrosis exhibit greater T-lineage reconstitution after irradiation and BMT by the hepatic portal vein, which recapitulates the clinical observations in AIDS patients with liver cirrhosis who received autologous BMT via hepatic portal vein.

The remarkable T-cell reconstitution by BMT via hepatic portal vein in AIDS patients with liver cirrhosis indicates that the direct traffic of T-cell progenitors from bone marrow to thymus is probably blocked. It appears that the hepatic portal vein and the fibrotic liver may have provided an alternate route and a nurturing niche, respectively, for the T progenitors. The fibrotic liver microenvironment in adult AIDS patients may have reactivated HSCs and directed their T-cell lineage development. Although there is no direct evidence that HIV-1 infection may reduce the seeding of T progenitors to thymus, it has been reported that the common lymphoid progenitors (CLPs) with reduced CCR7 exhibited impairment in their seeding in thymus^29^. Hematopoietic progenitor cells have been shown to have the potential to generate reservoirs for HIV, and infection and replication of HIV-1 were detected in purified T-cell progenitors of normal human bone marrow^30,31^. HIV-1 accessory protein Vpu downregulates CCR7 on the surface of CD4^+^ T cells^32^. Future studies may test whether HIV-1 infection can block T-cell-progenitor seeding to the thymus by downregulating CCR7 expression on CLPs.

Previous studies have shown that coculture of HSCs with OP9 bone marrow stromal cells overexpressing Notch ligands Dll4 enabled T-cell lineage generation in vitro^21^, and there was also report of induction of T-cell development by Dll4-expressing fibroblasts from mouse ear epidermal tissue^33^. The transcription factor FOXN1 was critically required for the development of thymic epithelial cells, and Dll4 was one of the target genes of Foxn1^34–36^. Enforced Foxn1 expression was sufficient to reprogram primary mouse embryonic fibroblasts into functional TECs; these Foxn1 induced TECs upregulated Dll4 expression and supported development of both CD4^+^ and CD8^+^ T cells in vitro^37^. We here show that primary LF with Dll4 OE could also promote T-cell development, but would lose this property after five passages. Hepatocyte cell line AML12 ectopically expressing Dll4 (AML12-Dll4) failed to support T-lineage development, but when mixed with primary LF they could promote T-cell development, indicating that in fibrotic liver microenvironment hepatocytes may activate Notch1 on HSCs' surface, by virtue of the upregulated Dll4, and liver fibroblasts could provide the other factors and cytokines necessary for T-lineage development. When DN2 and DN3 T-cell progenitors generated from HSCs/AML12-Dll4/LF cultures were transferred into immunodeficient Rag2^−/−^ mice, they differentiated into mature T cells that can be detected in the peripheral blood of the host.

Previous studies showed that the SDF-1/CXCR4 chemokine system was not required for T-cell development in embryos, but was involved in adult T-cell development^38–43^. SDF-1/CXCR4 also played an important role in the migration of T-cell precursors during development in both fetal and adult thymus^44–48^. Interestingly, SDF-1 is also a transcriptional target of Foxn1^36^. In the current study, we found that the promotion of T-cell development by LF-Dll4 was abolished when the SDF-1-CXCR4 signaling pathway was blocked or SDF-1 was downregulated by siRNA. SDF-1 was shown to be highly expressed in cells that support T-cell development, including OP9, TSC, and primary LF, but expressed at lower levels in cells that lack T-cell supportive effect, including P5 LF, NIH/3T3, MSC, AML12, and primary hepatocytes. We also show that SDF-1 expression in primary LF was rapidly downregulated after five passages. These results provided the evidence for the critical role of SDF-1-CXCR4 axis in T-lineage development from HSCs. In summary, we show that liver fibrosis could promote T-cell development by upregulating Dll4 and SDF-1 in liver tissues. This study has provided a mechanistic insight into the clinical efficacy of autologous BMT via hepatic portal vein in patients with AIDS and decompensated liver cirrhosis. It expanded our knowledge of the extrathymic T-cell development under pathological conditions.

## Materials and methods

### Mice

CD45.2/C57BL/6J, CD45.1/C57BL/6J, and *Rag2*^*−/−*^ mice were purchased from the Shanghai Laboratory Animal Center of Chinese Academy of Sciences, Shanghai, China, and maintained under specific pathogen-free conditions. Mice were maintained in the vivarium of Shanghai Institutes for Biological Sciences. Animals were matched for age and gender in each experiment. The animal protocols for the experiments described in this paper were approved by the Institutional Animal Care and Use Committee of the Institute of Health Sciences, Shanghai Institutes for Biological Sciences of Chinese Academy of Sciences.

### Reagents

Dulbecco’s modified eagle medium (DMEM), William’s E Medium, Minimum Essential Medium Eagle Alpha (αMEM), Glutamax, and penicillin/streptomycin were purchased from Invitrogen (Carlsbad, CA, USA). Fetal bovine serum (FBS) was from Thermo Fisher Scientific (Waltham, MA, USA). Anti-mouse CD25-coated and CD140a (PDGFRα)-coated magnetic microbeads were from Miltenyi Biotec (Miltenyi Biotec, Gladbach, Germany). Liver Dissociation Kit and Lineage Cell Depletion Kit were also purchased from Miltenyi Biotec (Miltenyi Biotec, Gladbach, Germany). Insulin-Transferrin-Selenium Ethanolamine Solution (ITS-X 100×) was purchased from Basal Media (Shanghai, China). NFκB inhibitor JSH-23 was purchased from Selleck (Houston, TX, USA). Recombinant Dll4 protein, TNFαR specific blocking antibodies, and anti-CD31, anti-αSMA, anti-CK18 antibodies for immunohistochemistry were from Abcam (Cambridge, UK). Anti-Dll4 antibodies for immunohistochemistry were from R&D system (Minneapolis, MN, USA). Donkey anti-goat IgG secondary antibody conjugated to Alexa Fluor 488 and goat anti-rabbit IgG secondary antibody conjugated to Cy3 were from Invitrogen (Carlsbad, CA, USA). Biotinylated anti-goat IgG secondary antibody and biotinylated anti-rabbit antibody were from MultiSciences (Hangzhou, China). VECTASTAIN ABC kit and DAB Peroxidase Substrate kit were purchased from vector labs (Burlingame, CA, USA). Bovine serum albumin (BSA), *N*-[*N*-(3,5-difluorophenacetyl-L-alanyl)]-S-phenylglycine t-butyl ester (DAPT), AMD3100, enzyme collagenase IV, dexamethasone, CsCl, and puromycin were purchased from Sigma-Aldrich (St. Louis, MO, USA). Mouse IL-7 and SCF were purchased from Peprotech (Rochy Hill, NJ, USA). Mouse Flt3L and mouse TNFα were from R&D system (Minneapolis, MN, USA). RNAprep pure Cell/Bacteria Kit and EndoFree Maxi Plasmid Kit was from Tiangen Biotech (Beijing, China). Primescript RT master Mix and PrimeSTAR^®^ GXL DNA Polymerase were from TAKARA (Kusatsu, Shiga Prefecture, Japan). SYBR Green Master Mix was from Roche Diagnostics (Indianapolis, IN, USA). QIAquick Gel extraction kit was from QIAGEN (Hilden, Germany). Coli DH5α and TransIT^®^-2020 Transfection Reagent were from Invitrogen (USA). KpnI and BamHI restriction enzymes were purchased from NEB (Ipswich, MA, USA). In-Fusion^®^ HD Cloning Kit was from Clontech (Mountain View, CA, USA). The kit of Dual-Luciferase^®^ Reporter Assay System was purchased from Promega (Madison, WI, USA). The fluorochrome-labeled antibodies (for flow cytometry) were purchased from Biolegend (San Diego, CA, USA).

### Cells

Cell lines of OP9, AML12, and NIH/3T3 were obtained from the Typical Model Cultivation Center of Chinese Academy of Sciences (Shanghai, China). TSCs were derived from thymus of C57BL/6 mice^23^. Mouse MSCs were generated from tibia and femur bone marrow using 6–10-week-old mice and were used from10th to 15th passage.

Primary mouse liver fibroblasts and P5 mouse liver fibroblasts were prepared as follows: livers were removed from euthanized C57BL/6J mice and were processed to generate single-cell suspensions by Liver Dissociation Kit (Miltenyi Biotec, Germany). Cell suspension was passed through 70 μm nylon cell strainer (FALCON, USA) and parenchymal mouse liver cells were removed by centrifugation and cells were then resuspended in the MACS buffer [Phosphate-buffered saline (PBS) pH 7.2, and 0.5% BSA, 1 mM EDTA]. CD140a (PDGFRα) MicroBead Kit was used to magnetically separate CD140a^+^ cells and resulting cells were cultured in DMEM [supplemented with 10% FBS, 2 mM Glutamax, 2 mM 2-mercaptoethanol, and penicillin (100 U/ml)/streptomycin (100 mg/ml)]. Cells were allowed to grow for 10 days to establish a cell culture of primary LF.

Primary hepatocytes were isolated by two-step collagenase perfusion of normal C57BL/6J liver. In brief: 30 ml EGTA buffer was perfused through the portal vein of anesthetized mice and then changed to 40 ml collagenase solution (enzyme collagenase IV 0.75 mg/ml in PBS) perfused in for ~5 min. The entire liver was removed and transferred to a Petri dish containing collagenase solution (enzyme collagenase IV 0.08 mg/ml in PBS) at 37 °C 100 rpm 5 min. The gallbladder was cut out and the liver lobes were separated using two pairs of forceps. The crude hepatocytes preparation was filtered through a gauze mesh filter (100 μm in diameter) and the resulting cell suspension was transferred into two 50-ml sterile tubes and centrifuged at 50 × g for 3 min. The cell pellets were washed three to four times in PBS by centrifuging at 50 g for 2 min. The cells were resuspended in William’s E Medium [supplemented with 10% FBS, 1 × Insulin-Transferrin-Selenium Ethanolamine Solution (ITS-X 100×), 40 ng/ml dexamethasone, 2 mM Glutamax, and penicillin (100 U/ml)/streptomycin (100 mg/ml)]. The cells were inoculated into 35 mm tissue culture dishes at 6 × 10^5^ of hepatocytes suspension per dish. After 6–8 h, the supernatant and unattached cells were discarded, and the adherent cells were then fed with fresh William’s E Medium. Cells culture continued as required.

### CCl_4_ treatment and BMT

Six-week-old male CD45.2/C57BL6/J mice were treated with 10 mL/kg CCl_4_ dissolved in corn oil (1:5) twice a week for 8 weeks. Mice were subjected to 6 Gy sublethal irradiation 24 h before BMT (3 × 10^6^ BMCs) from CD45.1/C57BL6/J mice in 200 μl PBS were injected into spleen to allow liver entry through the hepatic portal vein). Mice were continually treated with CCl_4_ for 4 weeks and were then euthanized to assess the extent of liver fibrosis. Peripheral blood, spleen, and thymus were collected to assess the stages of T-cell development.

### Preparation of BMCs

For BMCs isolation, CD45.1/C57BL6/J congenic mice (6-week-old) were euthanized by cervical dislocation followed by limbs removal. BMCs from CD45.1/C57BL6/J mice were flushed with DMEM containing 10% FBS from the medullary cavities of tibias and femurs using a 25-G needle.

### Immunohistochemistry

Frozen sections of mouse liver were subjected to immunohistochemical staining using anti-CD31, anti-αSMA, anti-CK18, and anti-Dll4 antibodies. In brief, sections were washed three times and treated with 0.1% trypsin for antigen unmasking and 3% H_2_O_2_ to block endogenous peroxidase activity. Sections were then blocked with reagent containing 5% BSA in PBS for 1 h. Goat polyclonal anti-Dll4 antibody (R&D; AF1389-SP) was used at 1:100 dilution, coupled with donkey anti-goat IgG secondary antibody conjugated to Cy3 at 1:500 dilution. Rabbit polyclonal anti-αSMA antibody (Abcam; ab5694) was used at 1:200 dilution, rabbit polyclonal anti-CD31 antibody (Abcam; ab28364) was used at 1:50 dilution, and rabbit polyclonal anti-CK18 (Abcam; ab24561) was used at 1:200 dilution, coupled with goat anti-rabbit IgG secondary antibody conjugated to Alexa Fluor 488 at 1:1000 dilution. Nuclei were stained using DAPI (Sigma; D9542).

Immunohistochemical staining of serial sections of human cirrhotic liver samples in paraffin, obtained from Shanghai Public Health Clinical Center, was performed using antibodies to CD31, αSMA, CK18, and Dll4. Goat polyclonal anti-Dll4 antibody (R&D; AF1389-SP) was used at 1:50 dilution, coupled with biotinylated anti-goat IgG secondary antibody at 1:500 dilution. Rabbit polyclonal anti-αSMA antibody (Abcam; ab5694) was used at 1:250 dilution, rabbit polyclonal anti-CD31 antibody (Abcam; ab28365) was used at 1:50 dilution, and rabbit polyclonal anti-CK18 (Abcam; ab24561) was used at 1:500 dilution, coupled with biotinylated anti-rabbit antibody at 1:500 dilution. After antibody staining, sections were washed in PBST, treated with VECTASTAIN ABC kit for biotin-streptavidin signal amplification, and subsequently visualized by DAB Peroxidase Substrate kit and nuclei were stained by hematoxylin stain.

### Adenovirus-mediated Dll4 OE in liver

The AdEasyTM adenoviral vector system was utilized to construct the adenovirus expression vectors^49^. For Dll4 expression, the encoding sequences were subcloned into pTrack-CMV vector and recombined with pAdEasy vector. The adenoviruses were packaged in HEK293A cells and purified with CsCl ultracentrifugation. The viruses were titrated and administered via caudal vein injection (10^9^ pfu viruses per mouse). Four days later, mouse liver tissues were collected and the level of Dll4 expression was examined by qRT-PCR and immunohistochemistry.

### Generation of Dll4-overexpressing cells

OP9, MSC, NIH/3T3, TSC, AML12, and mouse liver fibroblasts were transduced to express Dll4, as described for the OP9-DL1 and OP9-DL4 cells^50^. Briefly, the lentiviral vector pLVX-IRES-Puro containing the *Dll4* cDNA sequence was independently transfected into HEK-293T cells along with a plasmid pMD2.G and psPAX2 in DMEM. Lentiviral particles produced and released into the supernatant were used to infect the packaging cell line. Transfected cells were selected by puromycin at 1 μg/ml.

### Adenovirus particles for siRNA for SDF-1

SDF-1-specific and control siRNA adenovirus particles were prepared by GenePharma (Shanghai, China, http://www.genepharma.com).

### Flow cytometry

For the analysis of cell surface molecules on mouse cells, directly conjugated antibodies CD25-FITC, CD44-PE, and CD45.1-APC were used for analyzing the differentiation of HSCs cocultured with Dll4 OE cells on a GALLIOS flow cytometer (Beckman Coulter, Brea, CA, USA) using GALLIOS software.

For in vivo experiments, peripheral blood was collected from each mouse before euthanizing; spleen and thymus were also collected for lymphocyte staining. T-lymphocytic population changes were quantified by flow cytometry. Thymocytes were stained with antibodies to CD45.1-APC, CD45.2-Alex700, CD25-PE, and CD44-PerCP-Cy5.5, and a lineage-FITC mix containing antibodies to the following markers: CD4, CD8α, TCRβ, TCRγδ, CD19, NK1.1, DX5, Gr1 (Ly6C/G), CD11b, and Ter119 to facilitate gating and analyzing on stages of DN cells. To assess DP and SP cells, thymocytes were stained for CD45.1-APC, CD45.2-Alex700, CD3-PE, CD4-PerCP-Cy5.5, and CD8-FITC. Lymphocytes in peripheral blood and spleen were stained for CD45.1-APC, CD45.2-Alex700, CD3-PE, CD4-PerCP-Cy5.5, and CD8-FITC to assess different T-lymphocyte populations. To characterize Dll4, OE cells were stained with anti-Dll4-APC.

### Coculture of hematopoietic progenitor cells with Dll4 overexpressing cells

Hematopoietic progenitor cells were isolated from 8 weeks CD45.1/C57BL/6J BMCs. Lin^−^ bone marrow progenitor cells were enriched by Lineage Cell Depletion Kit and then FACS-sorted for the expression of CD117 and Sca-1 by flow cytometry. Mouse HSCs (1 × 10^5^/well in six-well tissue-culture plates, were incubated with different Dll4-overexpressing cells (semiconfluent) in αMEM [supplemented with 15% FBS, penicillin (100 U/ml), streptomycin (100 mg/ml), 10 ng/ml IL-7, 10 ng/ml Flt3L, and 50 ng/ml SCF]^22^. Cells were harvested on day 7 for analysis or further passaged onto fresh plates containing Dll4 overexpressing cells for analysis on days 10 and 13.

### Adoptive transfer of progenitor cells

Mouse HSCs were cocultured with AML-Dll4/primary LF in a 10-cm plate, in αMEM supplemented with 10 ng/ml IL-7, 10 ng/ml Flt3L, and 50 ng/ml SCF for 8 days. DN2 (CD44^+^CD25^+^) and DN3 (CD44^−^CD25^+^) cells from the coculture were sorted by CD25 MicroBeads, and 3–5 × 10^5^ DN2 cells were injected into the tail vein of 4–8-week-old *Rag2*^*−/−*^ mice. The peripheral blood of the injected mice was examined by flow cytometry for the presence of T cells on day 28 postinjection.

### qRT-PCR

Total RNA was isolated using RNAprep pure Cell/Bacteria Kit, and reverse transcribed into cDNA using primescript RT master Mix. Gene probes were used with SYBR Green Master Mix and a 7900HT Fast Real-Time PCR system (Applied Biosystems, Foster City, CA, USA). Total amount of mRNA was normalized to endogenous β-actin mRNA. Sequences of primer pairs were listed in Supplementary Table 1.

### Cloning of Dll4 promoter sequence

Genomic DNA was isolated from C57BL/6J primary hepatocytes by standard protocol. Using the GenBank sequence for mouse chromosome 2 as a template, we designed two steps PCR: first-step primers Dll4p-out-F: TTCCTCTCTCCTCCCTCAGC and Dll4p-Out-R: TGACGACGAAGGAGTTGGTG amplify 4499 bp of sequence from −3789 to + 710 bp of Dll4 transcriptional start site. The PCR products of the first step were used as templates for the second-step primer contained restriction enzyme site linkers as follows: Dll4-prom-KpnI-F: CGCGGTACCTTCTGCATAATGGGAGAAACTG and Dll4-prom-BamHI-R: CCGCGGGATCCCTCCACTCCGGGACTCCGAA amplify 3038 bp of sequence from −2728 to +310 bp of the Dll4 transcriptional start site. PCR was performed on a Tetrad 2 peltier thermal cycler (Bio-RAD, Hercules, CA, USA) using genomic DNA and PrimeSTAR^®^ GXL DNA Polymerase with the following parameters: 98 °C for 2 min (1 cycle), 98 °C for 10 s, 60 °C for 15 s, and 68 °C for 4 min 30 s in the first step, or 2 min 54 s in the second step (30 cycles). The second-step PCR products were electrophoretically separated on 0.8% agarose gels, and the appropriate-sized band cut out and purified using the QIAquick Gel extraction kit. Purified second-step PCR products were digested with KpnI and BamHI restriction enzymes and ligated into KpnI and BamHI-digested pGL3-basic Luciferase Reporter vector (Promega, USA). The putative NFκB binding site in the Dll4 promoter was mutated from (mutated bases bold and italicized) GGGAGTCTCC to ***TCT***AGTCTCC and GGGAGTCTCC to GGGAGTC***C***CC, using the In-Fusion^®^ HD Cloning Kit. All ligation reaction products were transformed into *E.coli* DH5α, and amplified and purified by EndoFree Maxi Plasmid Kit. The constructs were verified by sequencing (Sangon Biotech, Shanghai, China) and subsequent analysis using FinchTV 1.4 software (Geospiza Inc, Seattle, WA, USA). The putative transcription factor binding sites were identified using the TRANSFAC Database (www.gene-regulation.com).

### Transfections and luciferase reporter assays

Confluent C57BL/6J primary hepatocytes grown in 6-well or 10-cm plates were transfected according to the manufacturer’s instructions, with modifications using TransIT^®^-2020 Transfection Reagent. Briefly, 70–80% confluent C57BL/6J primary hepatocytes in 24-well plates were incubated with 0.5 ml transfection cocktail containing 500 ng pGL3-vector with the Dll4 promoter sequence and 50 ng Renila plasmid DNA per well. After 24 h, the transfection cocktail was replaced with fresh culture medium before 100 ng/ml TNFα treatment. Eight hours later, the cells were lysed and the reporter activities were calculated according to the protocol provided with the kit of Dual-Luciferase^®^ Reporter Assay System (Promega; E1910).

### Statistical analysis

Statistical analysis of all paired experiments was analyzed by two-tailed Student’s *t* test. All graphs were represented by mean and standard error of the mean, where n.s. = *P* > 0.05; *P < 0.05; **P < 0.01; ***P < 0.001; unless otherwise stated. All experiments were performed at least three times with similar results, except where indicated.

## Supplementary information

Legends to Supplementary Figures

supplementary figures 1

supplementary figures 2

supplementary figures 3

supplementary figures 4

supplementary figures 5

supplementary figures 6

supplementary figures 7

supplementary figures 8

supplementary figures 9

supplementary figures 10

supplementary figures 11

supplementary figures 12

Supplementary Table 1
