# Supplementary material for: Fibrotic liver microenvironment promotes Dll4 and SDF-1-dependent T-cell lineage development
Source: Cell Death Dis. 2019 Jun 5;10(6):440. doi: 10.1038/s41419-019-1630-1 (PMC6549170; doi:10.1038/s41419-019-1630-1)
Supplement: Supplementary file 1 — Legends to Supplementary Figures [file 41419_2019_1630_MOESM1_ESM.docx]

**Supplementary Fig. 1** CCl_4_ treatment did not affect CD45.1 T cell reconstitution in the liver after irradiation and BMT. (**a**) Flow cytometric analysis for the expression of CD4 and CD8 on lymphocytes for CD4^+^ and CD8^+^ T cells in the liver of C57BL/6J mice with or without CCl_4_ treatment on day 28 after CD45.1 BMT. The results are presented as mean ± S.E.M. Statistical significance was determined by Student’s t-test. Significance between samples is indicated in the figures as follows: n.s = P >0.05.

**Supplementary Fig. 2** CCl_4_ treatment did not affect CD45.2 T cell reconstitution after irradiation and BMT. (**a**) Flow cytometric analysis of the expression of CD25 and CD44 on thymocytes for DN1-DN4 stages of the T cell development in thymus on day 28 after CD45.1 BMT through the hepatic portal vein. (**b**) Flow cytometric analysis for the expression of CD4 and CD8 in thymocytes for the DP and SP stages of T cell development, on day 28 after CD45.1 BMT. (**c**) Flow cytometric analysis for the expression of CD4 and CD8 on PBMCs for CD4^+^ and CD8^+^ T cells in peripheral blood on day 28 after CD45.1 BMT. The results are presented as mean ± S.E.M. Statistical significance was determined by Student’s t-test. Significance between samples is indicated in the figures as follows: n.s = *P* >0.05.

**Supplementary Fig. 3** CCl_4_ treatment did not affect T cell development in the thymus. (**a**) Flow cytometric analysis for the expression of CD25 and CD44 on thymocytes at the DN1-DN4 stages of T cell development in the thymus of mice treated with or without CCl_4_ treatment. (**b**) Flow cytometric analysis of the expression of CD4 and CD8 on thymocytes at the DP and SP stages of T cell development in the thymus of mice treated with or without CCl_4_. The results are presented as mean ± S.E.M. Statistical significance was determined by Student’s t-test. Significance between samples is indicated in the figures as follows: n.s = *P* >0.05.

**Supplementary Fig. 4** Dll4 expression in fibrotic liver tissue. (**a**) The cell types and the level of Dll4 expression in fibrotic mouse liver tissue were examined by immunoﬂuorescence, using Dll4, CD31 and αSMA -specific antibodies. (**b**) Serial sections of liver tissue from different patients (A1-A3) suffering from AIDS and different degrees of liver cirrhosis. Immunohistochemistry analysis was used to examine the cell types with Dll4 expression and the level of Dll4 expression change in different patients, using Dll4, CD31 and αSMA -specific antibodies. (**c**) Mouse primary liver fibroblast and primary hepatocytes were isolated from CCl_4_ treated mice, mRNA was harvested for qRT-PCR analyzing Dll4 expression. The results are presented as mean ± S.E.M. Statistical significance was determined by Student’s t-test. Significance between samples is indicated in the figures as follows: *** *P* <0.001.

**Supplementary Fig. 5** Analysis of engraftment and differentiation of BMCs in control and Dll4 overexpression mice. (**a**) Flow cytometric analysis for the expression of CD25 and CD44 on thymocytes for DN1-DN4 stages of T cell development in thymus of control and Dll4 overexpression mice on day 28 after CD45.1 BMT. (**b**) Flow cytometric analysis for the expression of CD4 and CD8 in thymocytes for DP and SP stages of T cell development in the thymus of control mice and Dll4 overexpression mice on day 28 after CD45.1 BMT. (**c**) Flow cytometric analysis for the expression of CD4 and CD8 in lymphocytes for CD4^+^ and CD8^+^ T cells in peripheral blood and spleen of control mice and Dll4 overexpression mice on day 28 after CD45.1 BMT. The results are presented as mean ± S.E.M. Statistical significance was determined by Student’s t-test. Significance between samples is indicated in the figures as follows: n.s = P >0.05.

**Supplementary Fig. 6** qRT-PCR evaluation of Dll4 expression in thymus tissues of mice carrying Dll4 expression vectors (Dll4 OE) and empty vectors (NC). (**a**) qRT-PCR evaluation of Dll4 expression in thymus tissues of mice carrying Dll4 expression vectors (Dll4 OE) and empty vectors (NC). The result is presented as mean ± S.E.M. Statistical significance was determined by Student’s t-test. Significance between samples is indicated in the figures as follows: n.s = *P* >0.05.

**Supplementary Fig. 7** The TNFα concentrations in serum of CCl_4_ treated mice and control mice at different time points. (**a**) Serum TNFα concentrations in C57BL/6J mice at different time points of CCl_4_ treated and control were measured by ELISA, BMT was perform at 8 weeks after CCl_4_ treated. The results are presented as mean ± S.E.M. Statistical significance was determined by Student’s t-test. Significance between samples is indicated in the figures as follows: * *P* <0.05; ** *P* <0.01; *** *P* <0.001.

**Supplementary Fig. 8** Isolation of primary hepatocytes and their response to cytokine treatment. (**a**) Primary hepatocytes were successfully isolated by two-step collagenase perfusion of normal C57BL/6J liver tissues. (**b**) Primary hepatocytes were treated 12 h by the different cytokines and Dll4 mRNA expression was assessed by qRT-PCR.

**Supplementary Fig. 9** Assay of T lineage differentiation of HSCs co-cultured with different Dll4 expressing cell lines. (**a**) Sorted HSCs (Lin^-^Sca1^+^CD117^+^) derived from CD45.1/C57BL/6J mice bone marrow were co-cultured with OP9-Dll4 cells. HSCs were harvested after 0, 6,10 or 14 days and analyzed by ﬂow cytometry for the surface expression of CD25 and CD44 as indicated. Cells were gated as live (DAPI^-^) and CD45.1^+^. Data are representative of at least three independent experiments. (**b**) The expression of CD25 and CD44 were analyzed by flow cytometry on 0, 6 or 10 days after HSCs co-cultured with TSC-Dll4. (**c**) The upper panels show the expression of CD25 and CD44 analyzed by flow cytometry on days 7 after HSCs co-cultured with MSC, MSC-Dll4 or MSC-Dll4 (1:1 ratio) mixed with OP9, the below panels show the expression of CD25 and CD44 were analyzed by flow cytometry on days 7 after HSCs co-cultured with NIH/3T3, NIH/3T3-Dll4 or NIH/3T3-Dll4 (1:1 ratio) mixed with OP9.

**Supplementary Fig. 10** αSMA expression on primary and P5 liver fibroblasts. (**a**) Flow cytometric analysis of the expression of αSMA on primary liver fibroblasts and P5 liver fibroblasts.

**Supplementary Fig. 11** Dll4 expression on primary LF-Dll4 and primary hepatocytes with or without TNFα treated. (**a**) Flow cytometric analysis of the expression of Dll4 on primary hepatocytes (Ctrl), TNFα (100 ng/ml) treated primary hepatocytes (TNFα) and primary LF-Dll4 (Dll4 OE).

**Supplementary Fig.12** Dll4 signaling driven T lineage development. (**a**) Primary LF-Dll4 cells co-cultured with HSCs were treated with or without notch inhibitor DAPT (10μM), the expression of CD25 and CD44 were analyzed by flow cytometry after 7 days. (**b**) Primary LF cells were isolated from mouse control liver or fibrosis liver with or without Dll4 overexpression and co-cultured with HSCs, the expression of CD25 and CD44 were analyzed by flow cytometry after 7 days.
