# Supplementary figures and images for: Fibrotic liver microenvironment promotes Dll4 and SDF-1-dependent T-cell lineage development
Source: Cell Death Dis. 2019 Jun 5;10(6):440. doi: 10.1038/s41419-019-1630-1 (PMC6549170; doi:10.1038/s41419-019-1630-1)

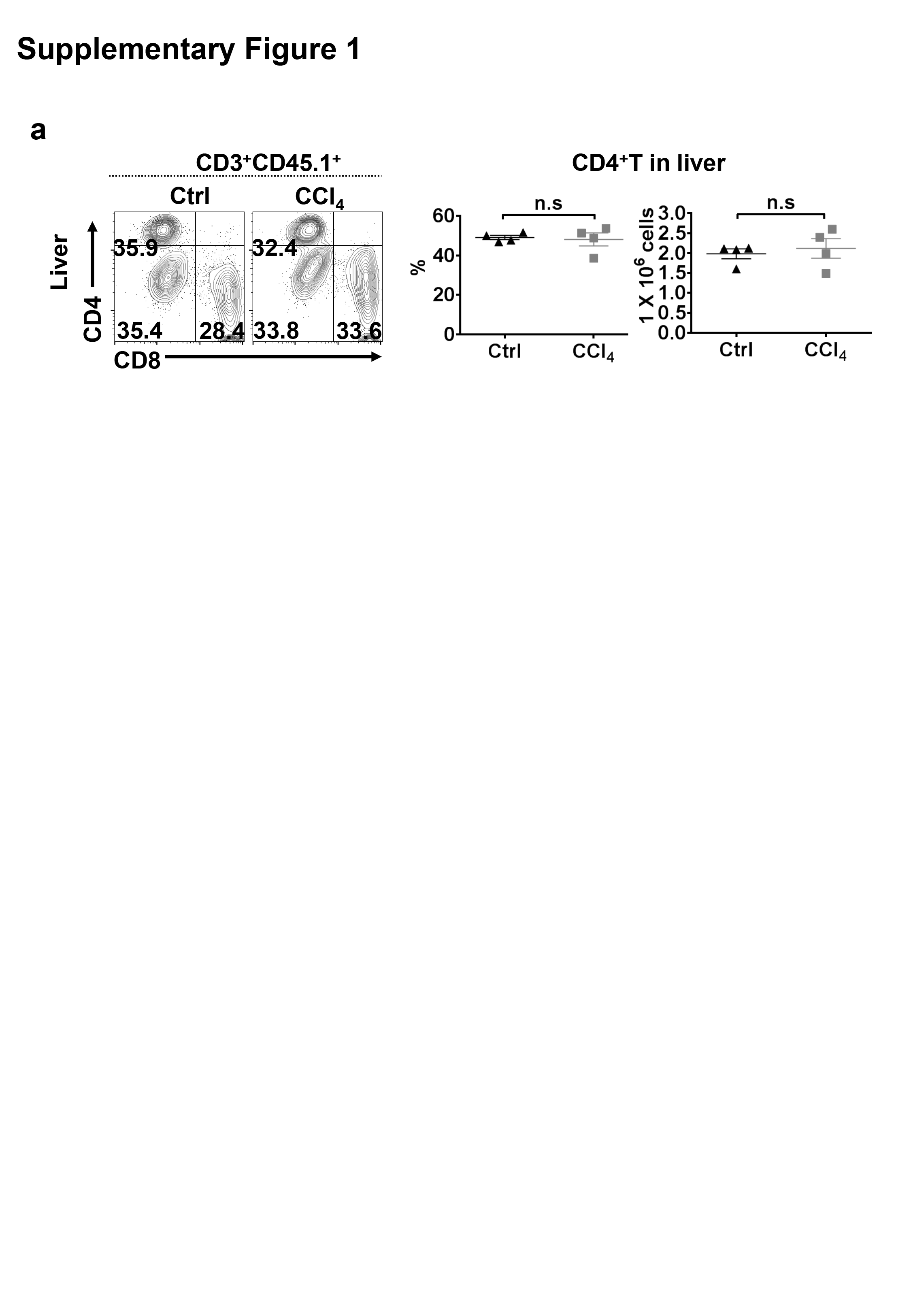

Supplement: Supplementary file 2 — supplementary figures 1 [file 41419_2019_1630_MOESM2_ESM.tif]

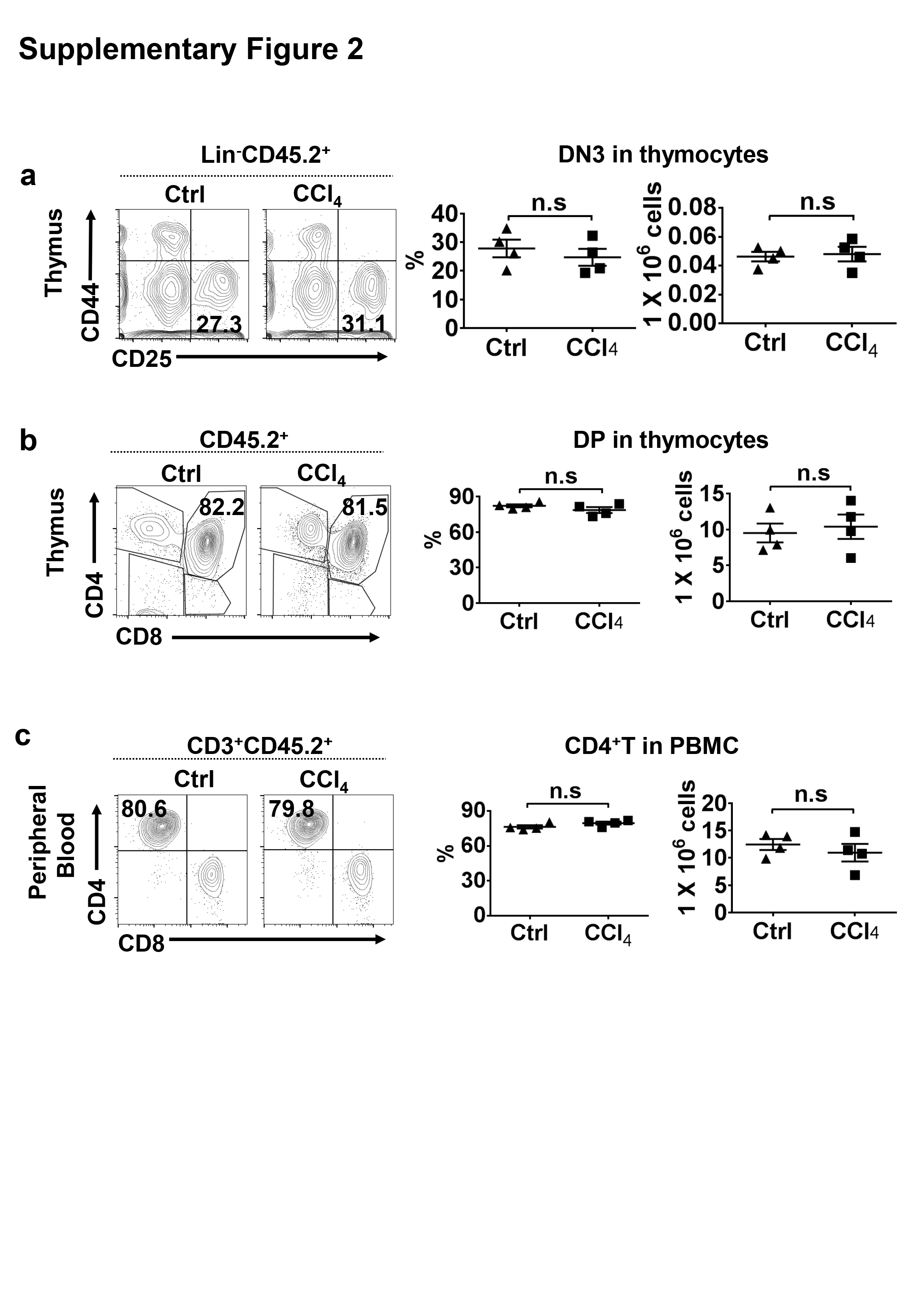

Supplement: Supplementary file 3 — supplementary figures 2 [file 41419_2019_1630_MOESM3_ESM.tif]

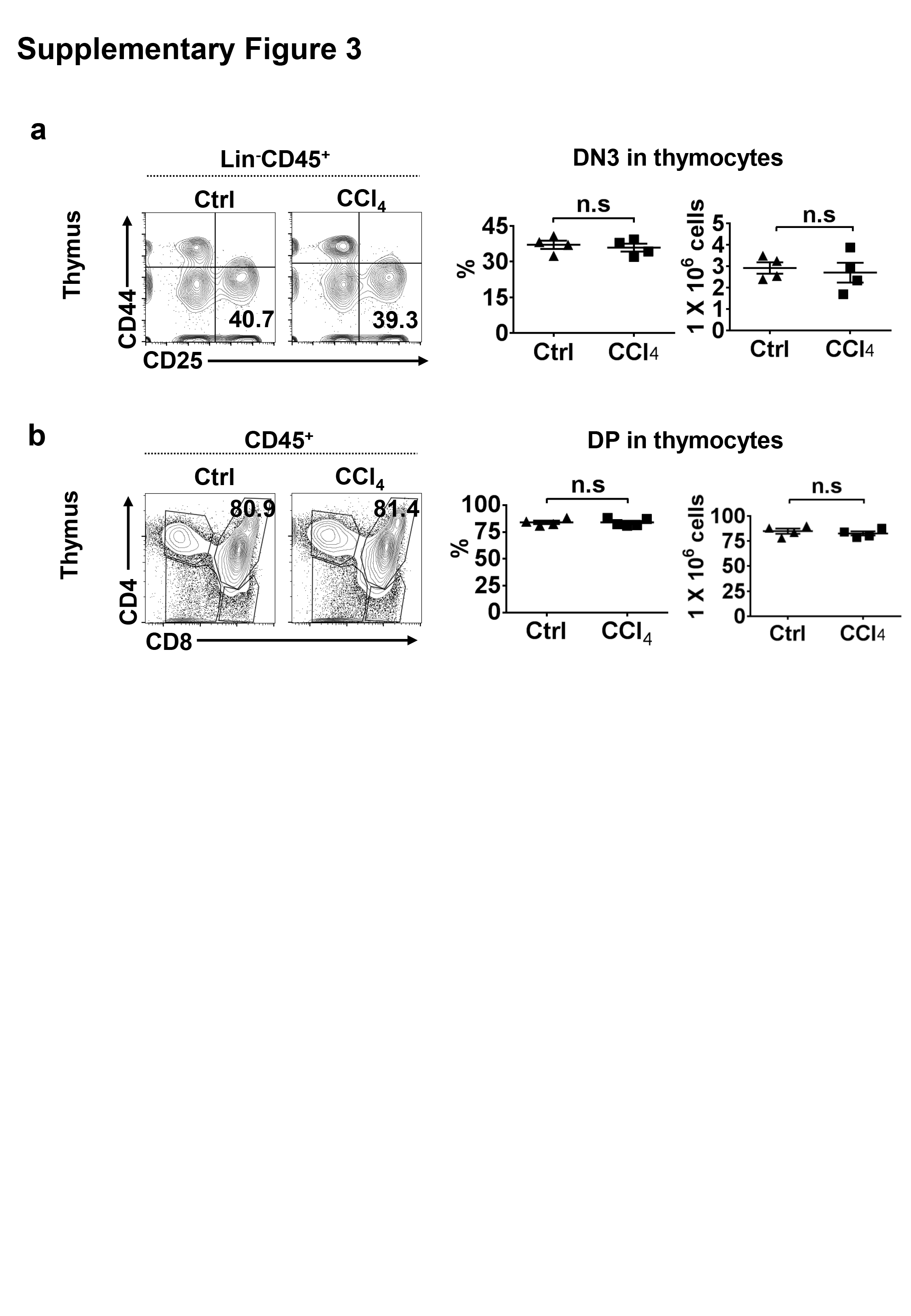

Supplement: Supplementary file 4 — supplementary figures 3 [file 41419_2019_1630_MOESM4_ESM.tif]

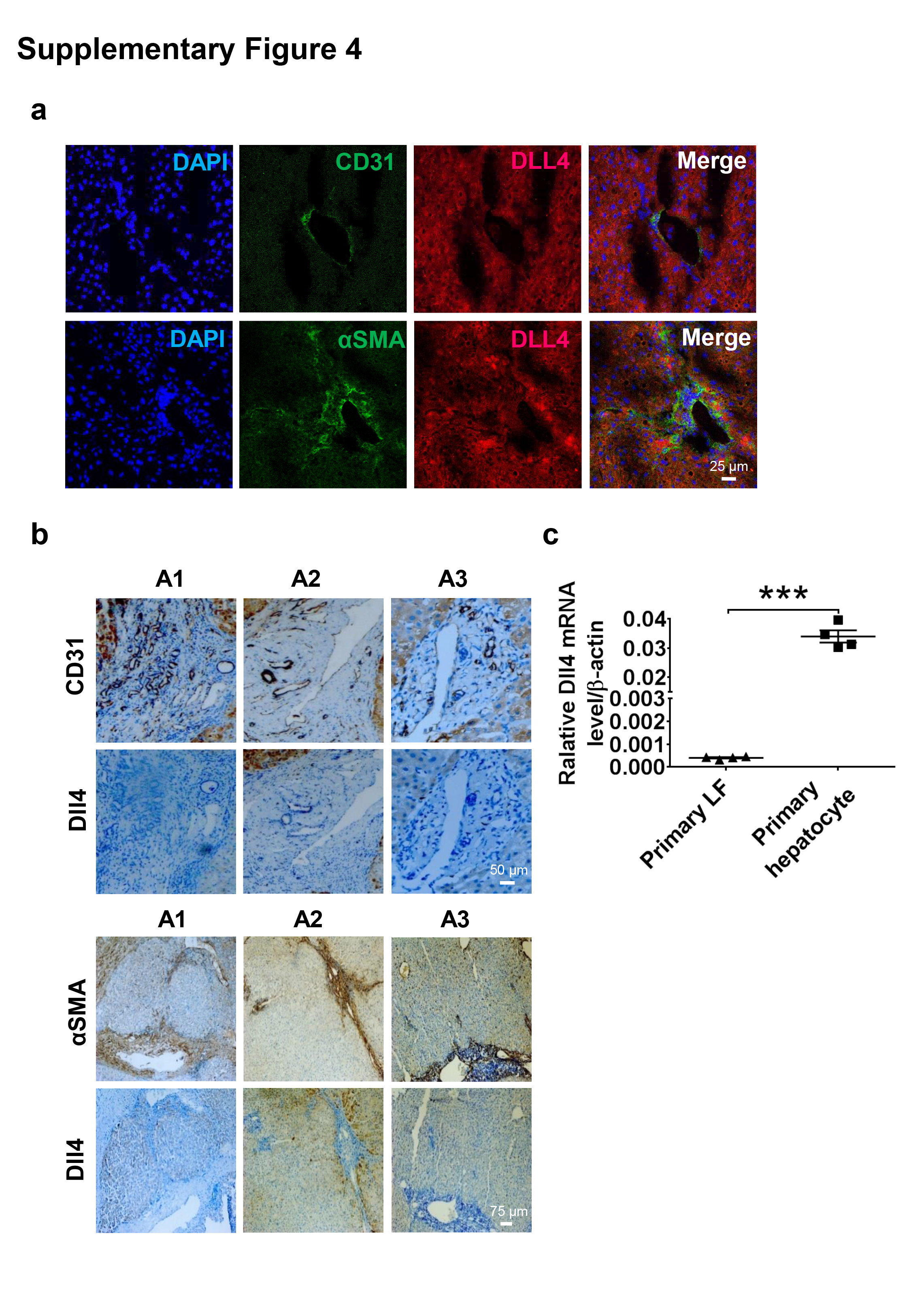

Supplement: Supplementary file 5 — supplementary figures 4 [file 41419_2019_1630_MOESM5_ESM.tif]

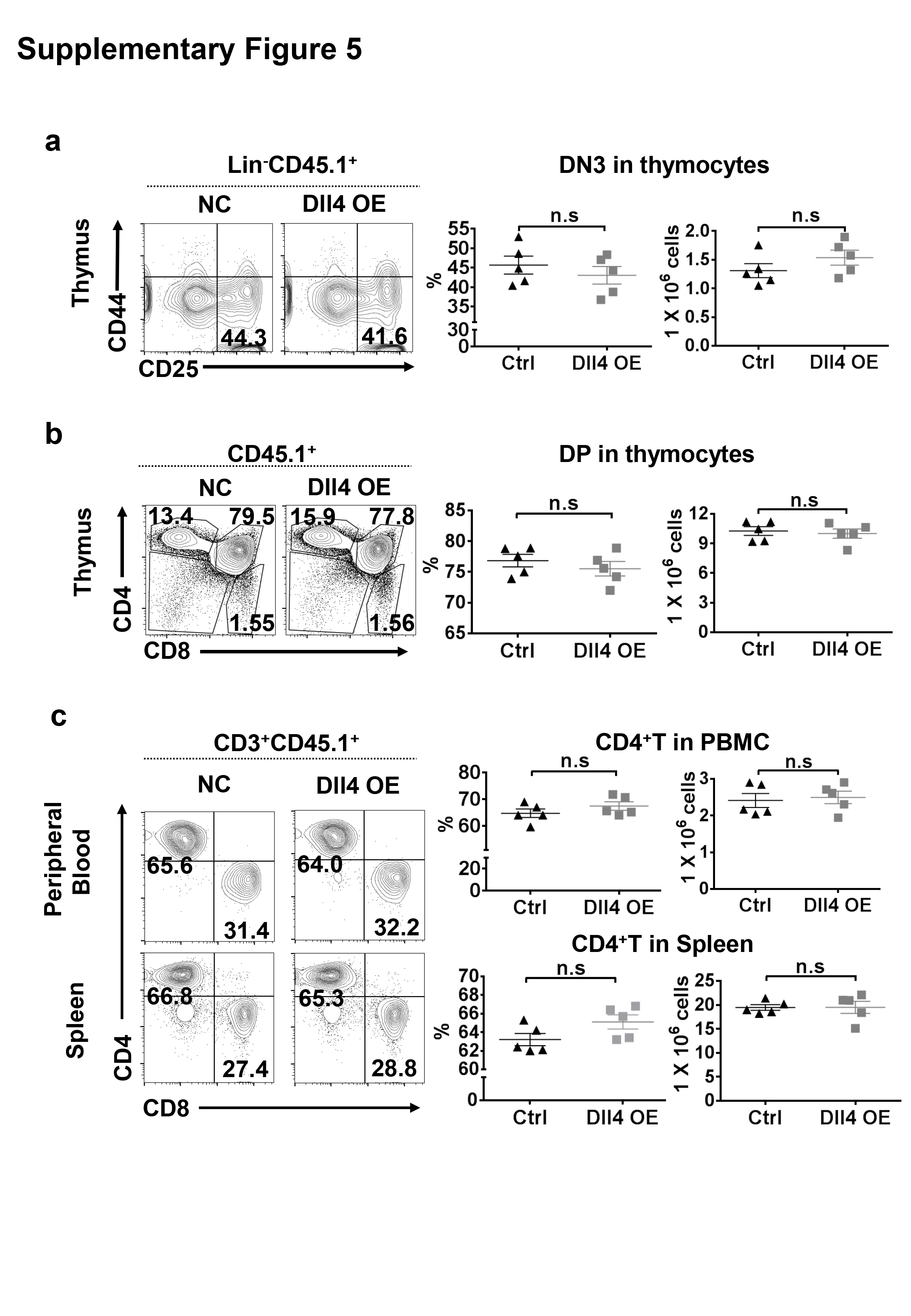

Supplement: Supplementary file 6 — supplementary figures 5 [file 41419_2019_1630_MOESM6_ESM.tif]

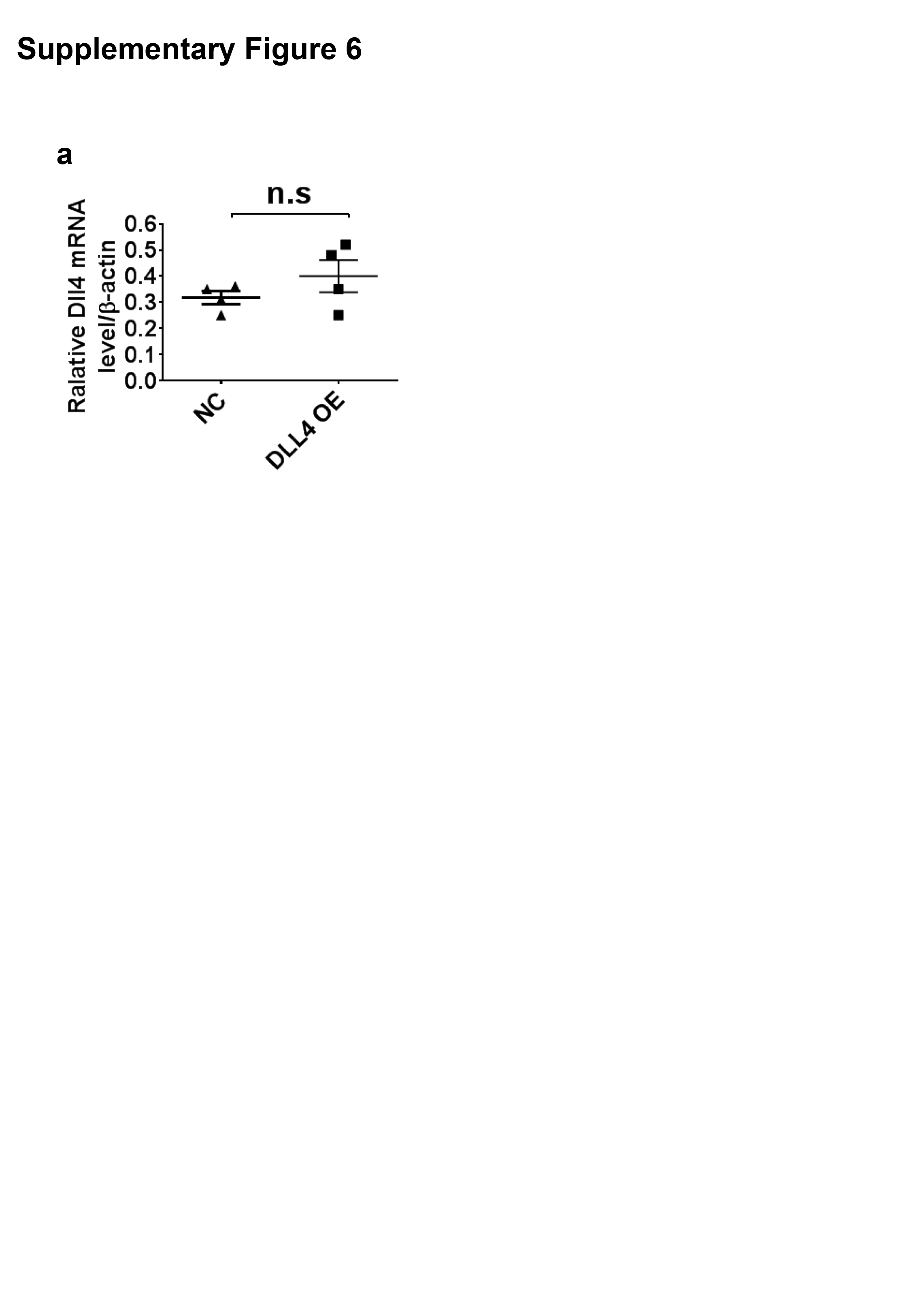

Supplement: Supplementary file 7 — supplementary figures 6 [file 41419_2019_1630_MOESM7_ESM.tif]

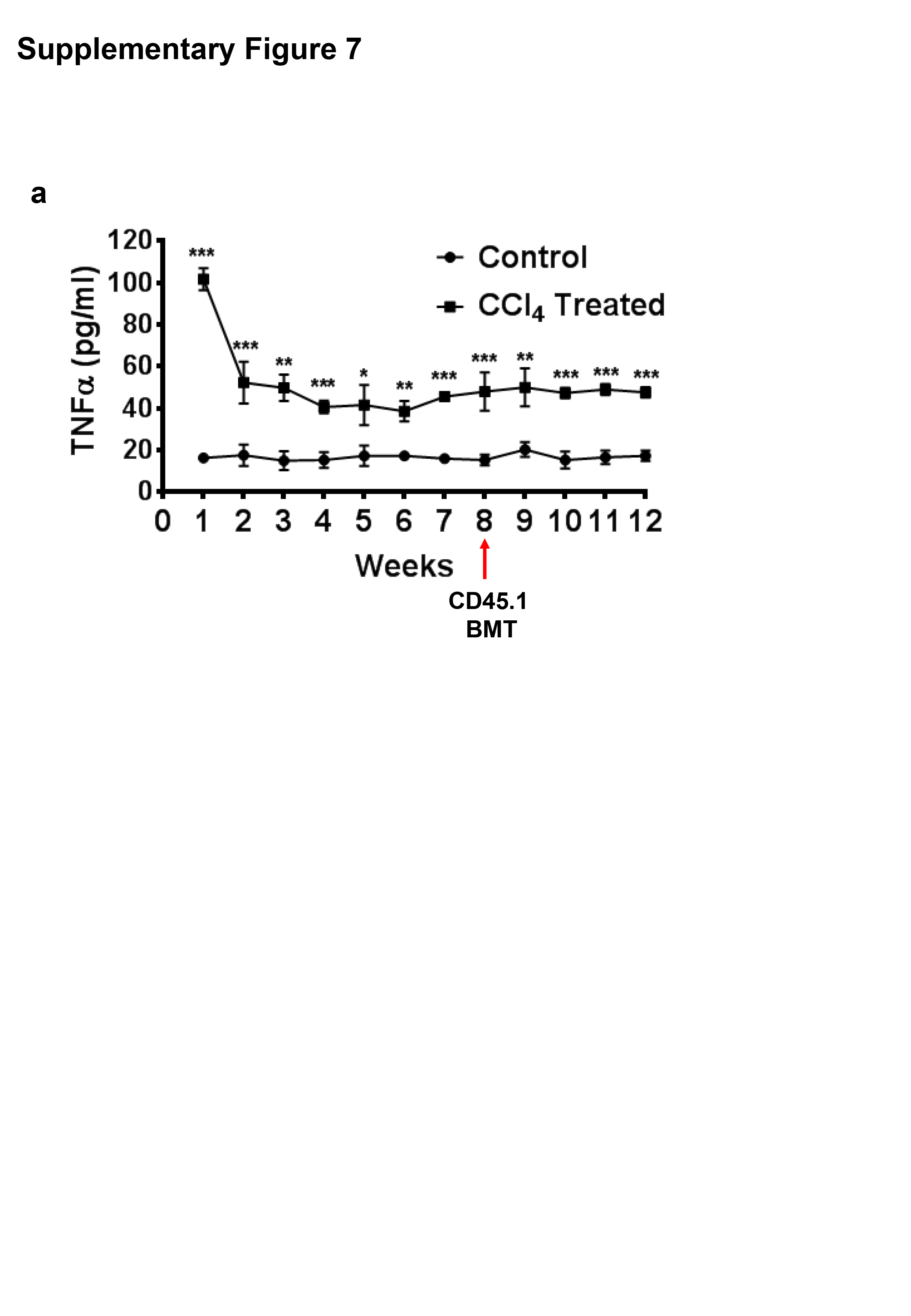

Supplement: Supplementary file 8 — supplementary figures 7 [file 41419_2019_1630_MOESM8_ESM.tif]

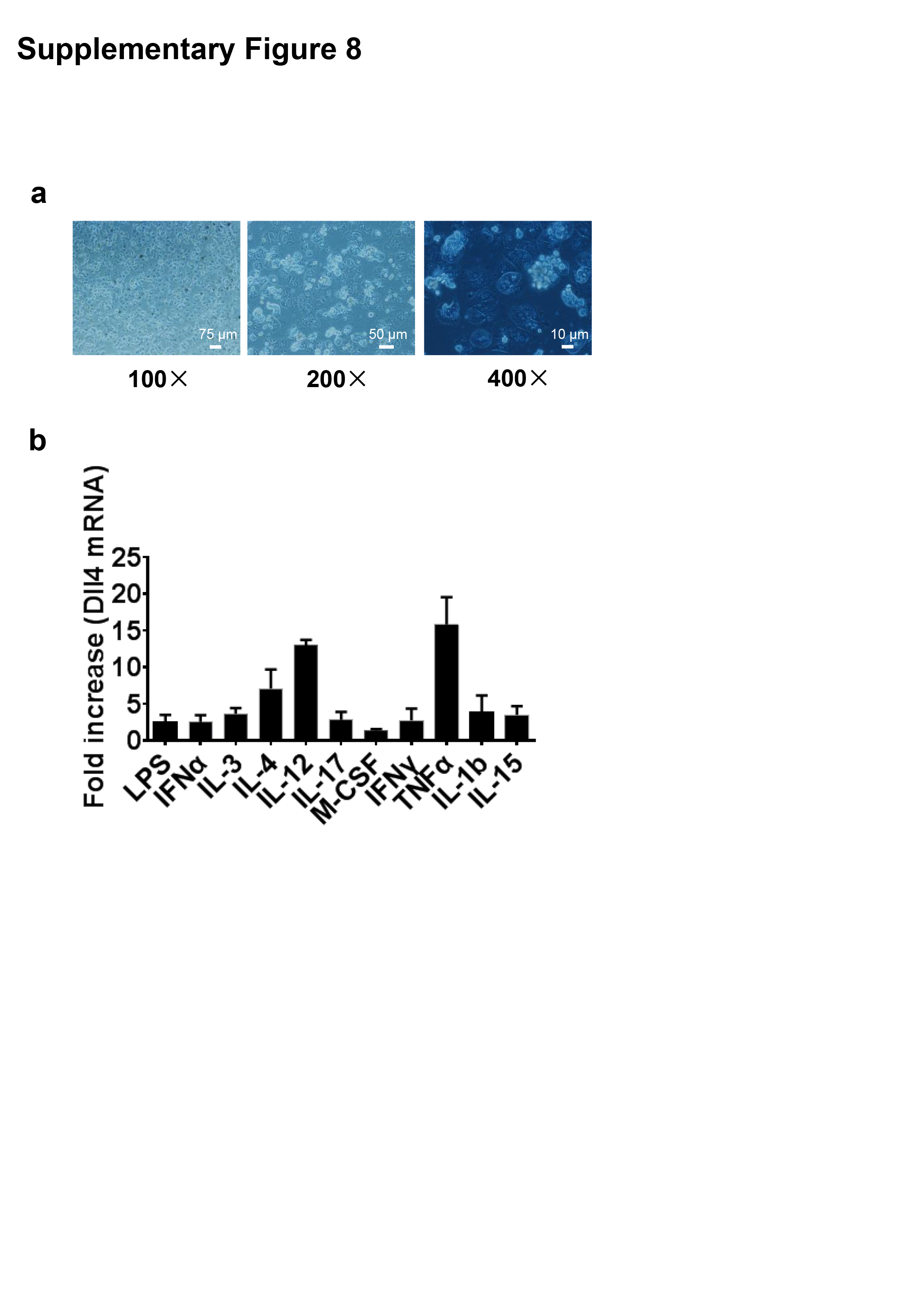

Supplement: Supplementary file 9 — supplementary figures 8 [file 41419_2019_1630_MOESM9_ESM.tif]

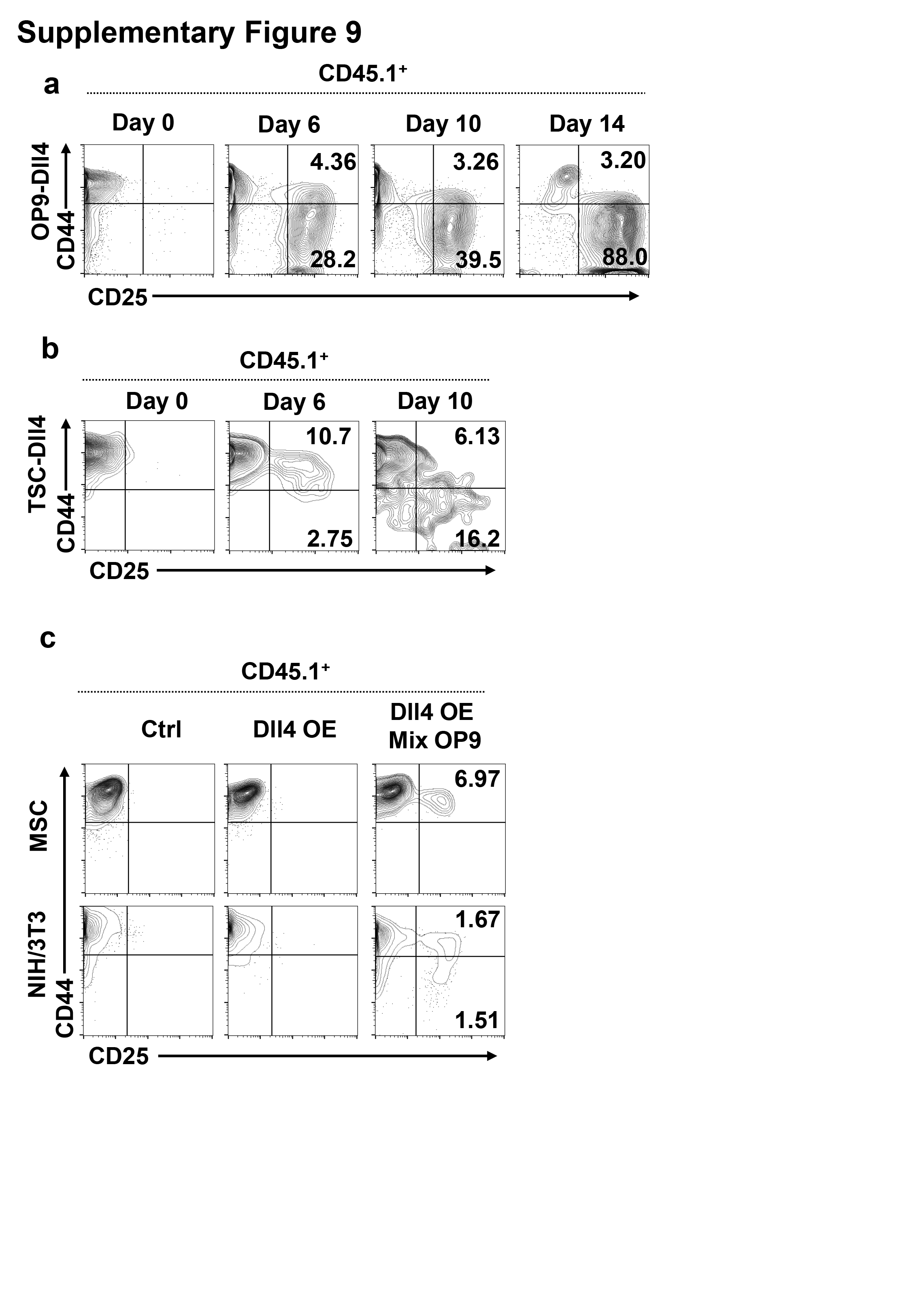

Supplement: Supplementary file 10 — supplementary figures 9 [file 41419_2019_1630_MOESM10_ESM.tif]

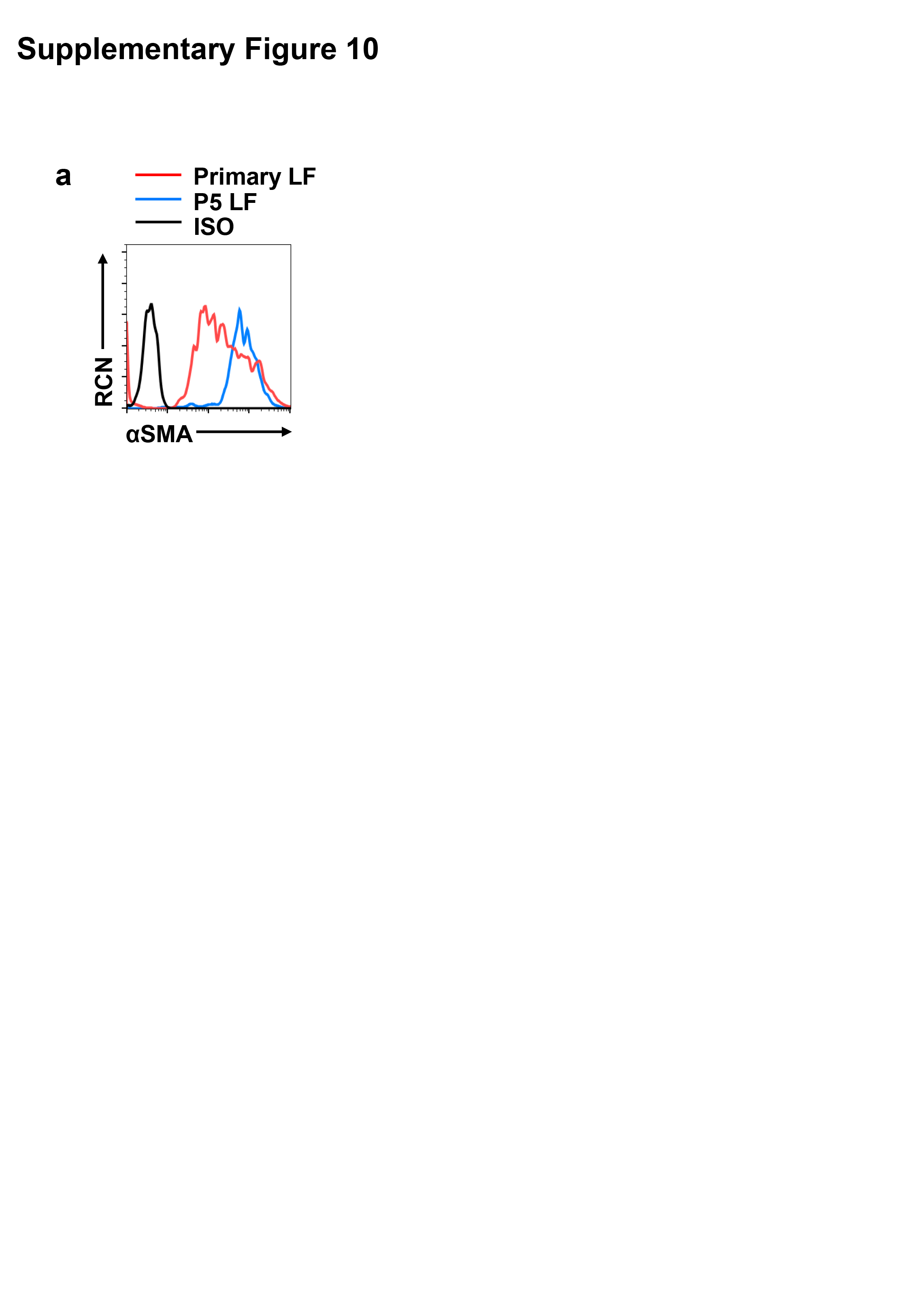

Supplement: Supplementary file 11 — supplementary figures 10 [file 41419_2019_1630_MOESM11_ESM.tif]

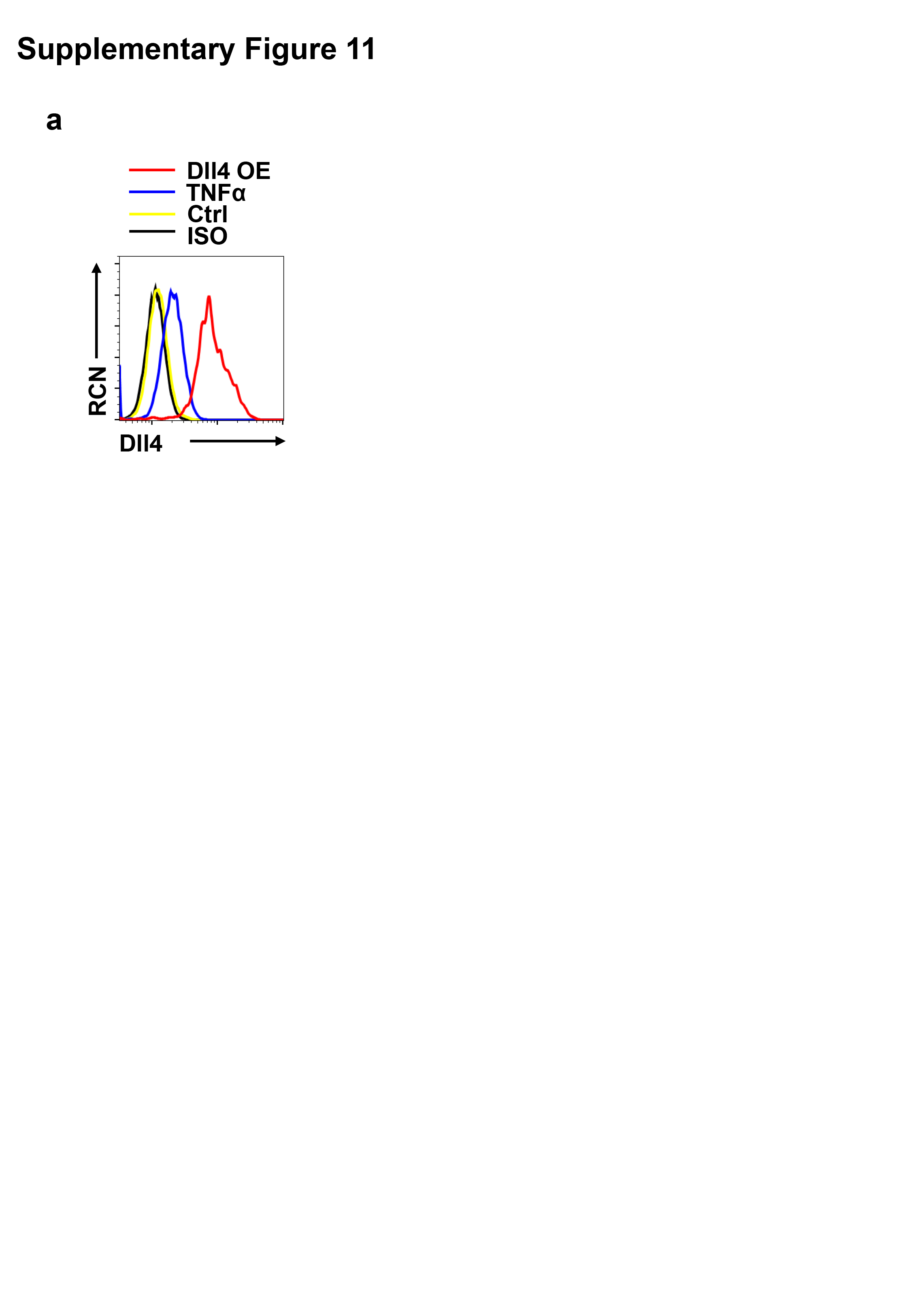

Supplement: Supplementary file 12 — supplementary figures 11 [file 41419_2019_1630_MOESM12_ESM.tif]

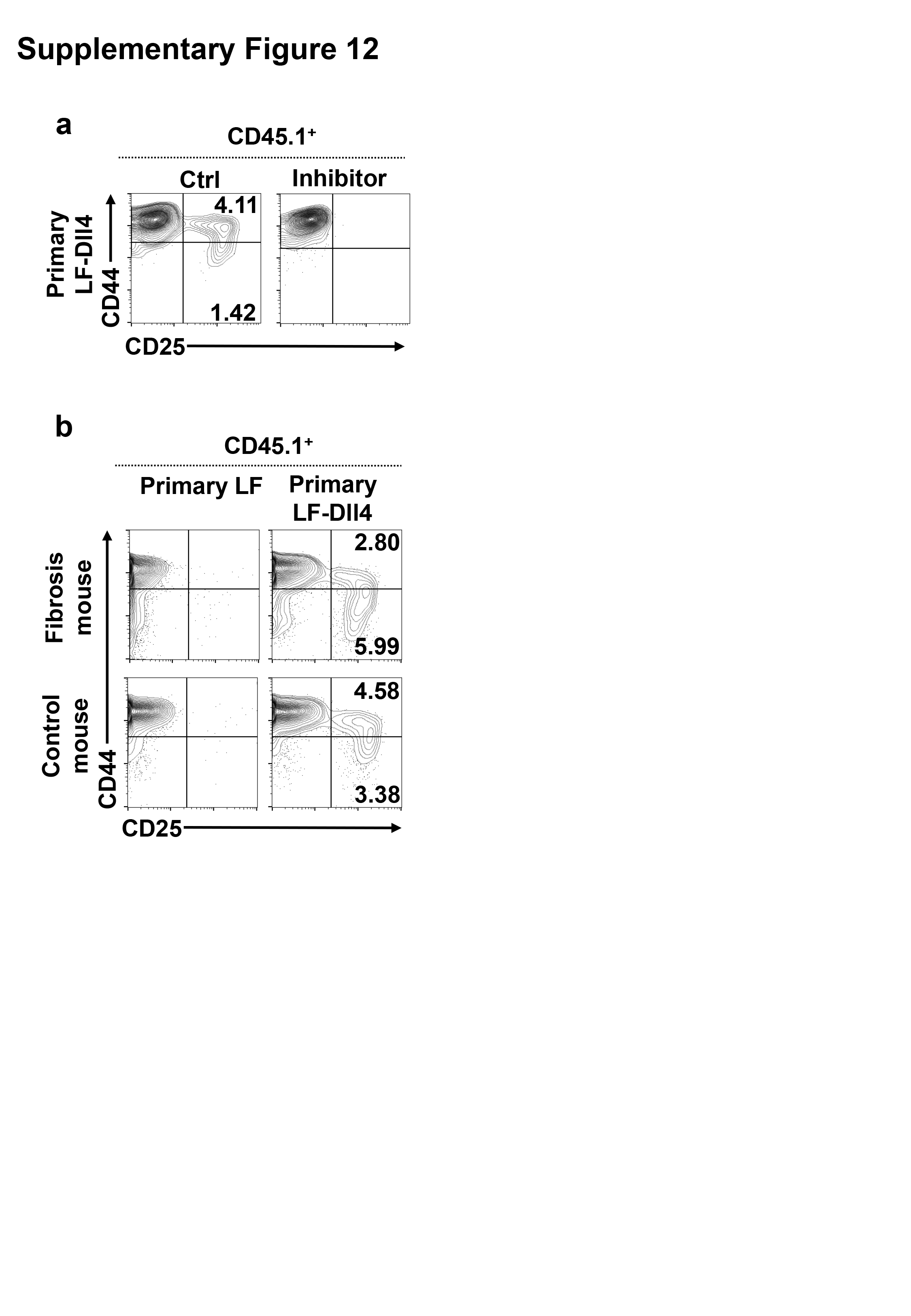

Supplement: Supplementary file 13 — supplementary figures 12 [file 41419_2019_1630_MOESM13_ESM.tif]
