# Supplementary material for: Fibrotic liver microenvironment promotes Dll4 and SDF-1-dependent T-cell lineage development
Source: Cell Death Dis. 2019 Jun 5;10(6):440. doi: 10.1038/s41419-019-1630-1 (PMC6549170; doi:10.1038/s41419-019-1630-1)
Supplement: Supplementary file 14 — Supplementary Table 1 [file 41419_2019_1630_MOESM14_ESM.docx]

**Supplementary Table 1**

|  | Forward | Reverse |
| --- | --- | --- |
| mouse |  |  |
| CXCL12 | TGCATCAGTGACGGTAAACCA | CACAGTTTGGAGTGTTGAGGAT |
| Dll4 | TTCCAGGCAACCTTCTCCGA | ACTGCCGCTATTCTTGTCCC |
| β-actin | GGCTGTATTCCCCTCCATCG | CCAGTTGGTAACAATGCCATGT |
